# Competition between cyclization and unusual Norrish type I and type II nitro-acyl migration pathways in the photouncaging of 1-acyl-7-nitroindoline revealed by computations

**DOI:** 10.1038/s41598-020-79701-4

**Published:** 2021-01-14

**Authors:** Pierpaolo Morgante, Charitha Guruge, Yannick P. Ouedraogo, Nasri Nesnas, Roberto Peverati

**Affiliations:** Chemistry Program, Florida Institute of Technology, 150 W. University Blvd, Melbourne, FL 32901 USA

**Keywords:** Organic chemistry, Photochemistry, Reaction kinetics and dynamics, Computational chemistry, Density functional theory

## Abstract

The 7-nitroindolinyl family of caging chromophores has received much attention in the past two decades. However, its uncaging mechanism is still not clearly understood. In this study, we performed state-of-the-art density functional theory calculations to unravel the photo-uncaging mechanism in its entirety, and we compared the probabilities of all plausible pathways. We found competition between a classical cyclization and an acyl migration pathway, and here we explain the electronic and steric reasons behind such competition. The migration mechanism possesses the characteristics of a combined Norrish type I and a 1,6-nitro-acyl variation of a Norrish type II mechanism, which is reported here for the first time. We also found negligible energetic differences in the uncaging mechanisms of the 4-methoxy-5,7-dinitroindolinyl (MDNI) cages and their mononitro analogues (MNI). We traced the experimentally observed improved quantum yields of MDNI to a higher population of the reactants in the triplet surface. This fact is supported by a more favorable intersystem crossing due to the availability of a higher number of triplet excited states with the correct symmetry in MDNI than in MNI. Our findings may pave the way for improved cage designs that possess higher quantum yields and a more efficient agonist release.

## Introduction

The ability to deliver or activate a compound to a specific site with precise timing is of extreme value. This is widely referred to as spatio-temporal control^1–4^. Techniques that possess spatio-temporal advantages have found numerous applications ranging from medicinal chemistry fields with photo targeted therapeutics^1,3^, such as cancer treatments^5–8^ or photoinitiated catalysis^8^, and to the neuroscience where it becomes critical to activate selected neurons to study specific pathways^1,9–12^. In the emerging field of optogenetics, light-sensitive channel rhodopsin receptors are genetically engineered in selected neurons rendering them light responsive^3,11,13–19^. Additional methods of firing specific neurons employed a diverse array of photocleavable protecting groups attached to critically active moieties of agonists^20–23^. These photocleavable protecting groups acquired the term photocages, or simply cages, as their attachment to an agonist necessarily incapacitates it, much like caging a ferocious animal.

**Figure 1:**
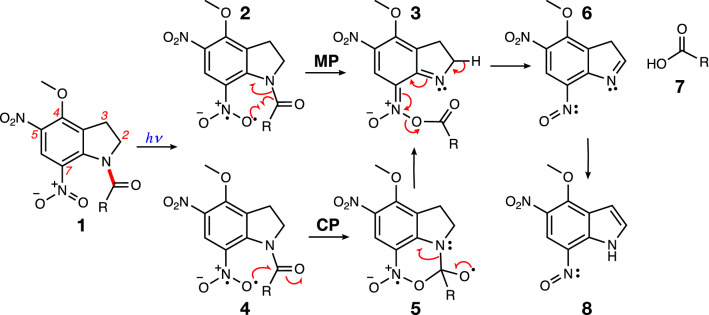
Different proposed mechanisms for the uncaging of MDNI-Glu (R=CH_2_CH_2_CH(NH_2_)COOH): Migration Pathway (MP)—computational mechanism reported by Pálfi et al.^39^; Cyclization Pathway (CP)—mechanism presumed by Ellis-Davies et al.^34^, based on Morrison’s experimental data^40^.

Caged molecules are inert, and they need an external agent—such as a physical, chemical, or mechanical force^8,24–27^—to induce the release of the biologically active compound they are protecting. Light represents the most advantageous activator because it offers the ability to control the precise time and the location of the release^1–4^. Although there are numerous classes of photocleavable groups, they share a common feature of absorbing light of specific wavelengths that leads to excitation from the singlet ground state (S_0_) to an excited singlet state (S_1,2,3…_). Subsequently, the molecules relax to the lowest excited singlet (S_1_) via internal conversion^28^, and some systems undergo intersystem crossing (ISC) to the triplet state manifold, and consequential fast relaxation to the lowest triplet state (T_1_)^21,29^. This leads to cleavage of the protecting group at the most labile bond, which is critically positioned for such release (displayed by the red bond of 4-methoxy-5,7-dinitroindolinyl glutamate (**1**, MDNI-Glu), shown in Fig. 1 below). These mechanisms depend on the class of protecting groups employed, but some are not clearly understood, as mechanisms in excited states may not resemble the familiar mechanistic pathways commonly accepted for ground states^21^.

7-Nitroindolinyl cages have gained popularity at the turn of the century due to their optimal quantum yields and efficient release of agonists^30–32^. Improvements in such cage systems led to the installation of a second nitro group at the 5 position that resulted in improved quantum yields and efficiency of the release^33,34^. Although the second nitro at the C-5 is not directly participating in the mechanism, as we will show later, its presence results in an improved quantum yield by at least fivefold according to experimental data^35–37^. The mechanism of uncaging for the 7-indolinyl system was studied by at least three groups using kinetic, fast pulse IR, and computation^33,38,39^. However, the most recently reported computational mechanisms of uncaging do not explain the difference in quantum yield, which is an important parameter, especially for consideration in future improved designs. We herein report a thorough computational analysis highlighting the electronic effects behind the better quantum yields of dinitroindolinyl systems and clarifying the mechanism with respect to the traditionally accepted formation of a cyclic intermediate.

The first mechanism for the light-initiated uncaging of MDNI-Glu (**1** in Fig. 1) has been proposed by Ellis-Davies and coworkers in 2005^34^, and is based on kinetic data collected by Morrison et al.^40^ for another member of the 7-nitroindoline family. We will refer to this mechanism as the cyclization pathway (CP) from here on for simplicity. According to their proposed mechanism, after irradiation, the reaction proceeds on the triplet surface via a cyclic intermediate (**5**). Subsequently, the system is deprotonated (**3**), and finally, it delivers the free glutamate (**7**) to the reaction medium. According to the CP mechanism, the increased reactivity of MDNI is due to the influence of the nitro group in position 5 on the overall electronic structure of the indoline scaffold^34^. A subsequent computational study by Pálfi et al. (the migration pathway, MP)^39^, however, did not report any cyclic intermediate, and it collected no evidence about a possible cyclization pathway. Also, Pálfi et al. attributed the increased reactivity of MDNI to a smaller difference in the enthalpy of activation (∆∆H) in one of the steps of the uncaging reaction on the triplet surface. The most notable difference between the proposed CP mechanism and the MP mechanism is the formation of the cyclic intermediate **5**, and to which extent the substituents of the indoline scaffold affect each pathway.

## Computational methods

In this work, we aim to redeem the controversy by performing state-of-the-art density functional theory (DFT) calculations of the full reaction mechanism with the ωB97X-D exchange–correlation functional approximation^41^ and the def2-TZVP basis set^42^ on top of geometries optimized at the B3LYP-D3(BJ)/def2-SVPD level of theory^42–46^. We chose the ωB97X-D method based on the fact that it reproduces the experimental UV–Vis spectra of both 4-methoxy-7-nitroindolyl (MNI) and MDNI (vide infra), and we validated its results using several other exchange–correlation functionals (ωB97M-V, M11, MN15, DSD-PBEP86-D3(BJ), LC-ωHPBE and CAM-B3LYP, see section S2 of the Supplementary Information for details). The B3LYP-D3(BJ)/def2-SVPD method was used for geometry optimization of all species because it was shown to produce reliable geometries for several systems in previous studies^47–49^. To address the potential multi-reference character of some of the species on the excited triplet surface, we also used the B_1_^50^ and A_λ_^51^ diagnostics, which showed a lack of strong multi-reference character for all the relevant transition structures (see sections S4 and S8 and Tables S1 and S2 in the Supplementary Information). We calculated and also report the Gibbs free energy profile of the reaction for the first time, including enthalpic and entropic corrections. All calculations have been performed in water using the Conductor-like Polarizable Continuum Model (C-PCM) framework^52,53^ (section S13 for more information). For computational efficiency, we first focused our attention on MDNI acetate (MDNI-Ac) and MNI acetate (MNI-Ac), two systems that closely resemble the original MDNI-Glu and MNI-Glu systems reported in the literature. Our results show competition between the cyclization and migration pathways, but they do not significantly differ from each other. The extension of the study to the relevant structures of the larger MDNI-Glu and MNI-Glu systems shows very little difference in the reactivity of the glutamate with respect to the acetate.

## Results and discussion

The cyclic intermediate proposed by Ellis-Davies indeed forms, and it represents the lowest-energy pathway in the overall reaction. However, the difference with the migration mechanism is negligible, ~ 2.6 kcal/mol for MDNI-Ac calculated at the ωB97X-D/def2-TZVP level of theory. Additional calculations on the MDNI-Glu and α-MDNI-Glu systems suggest that the different caged moieties do not affect the mechanism of the reaction on the triplet surface contrarily to what has been previously reported^39^. The cyclization and migration steps are within 2.3 kcal/mol from each other independently of how the glutamate is bound to the cage scaffold. This amount is very close to the one we obtained for MDNI-Ac, so we concluded that the uncaging mechanism is not affected by the length of the side chain, nor by its nature. Also, the second nitro group in position 5 does not affect the pathway significantly. The differences we found are within ~ 4.0 kcal/mol of each other (Table S3 in the Supplementary Information), and the highest activation energy for the migration pathway is 10.8 kcal/mol, while it is 8.2 kcal/mol for the cyclization pathway. Such barriers do not represent an obstacle to the reactivity of the molecule, as they are easily overcome at room temperature. Since we excluded any significant effect from the substituents to the uncaging mechanism, other factors may be contributing to the observed differences in quantum yields. For instance, the increased reaction quantum yield of the MDNI-based cages relative to their mononitro analogues could be attributed to a better photosensitivity due to the second nitro group. It has been shown that rates of ISC are increased upon substitution with heavy atoms (such as Br, I) or with functional groups that have low-lying n, π* states (e.g. carbonyls, nitro groups)^54^. As the reaction takes place in the triplet excited state, an improvement in the intersystem crossing of the excited singlet state of the molecule (carrying the additional nitro group) populates the triplet state more efficiently, and thus could be responsible for the overall improvement of the reaction, hence its quantum yield. We investigated this using time-dependent density functional theory (TD-DFT) calculations to analyze the electronic structure of the excited states manifold. This approach can be used to screen potential candidates for future applications, and it can help identify and select the compounds with the best light-absorbing and light-responsive properties.

**Figure 2:**
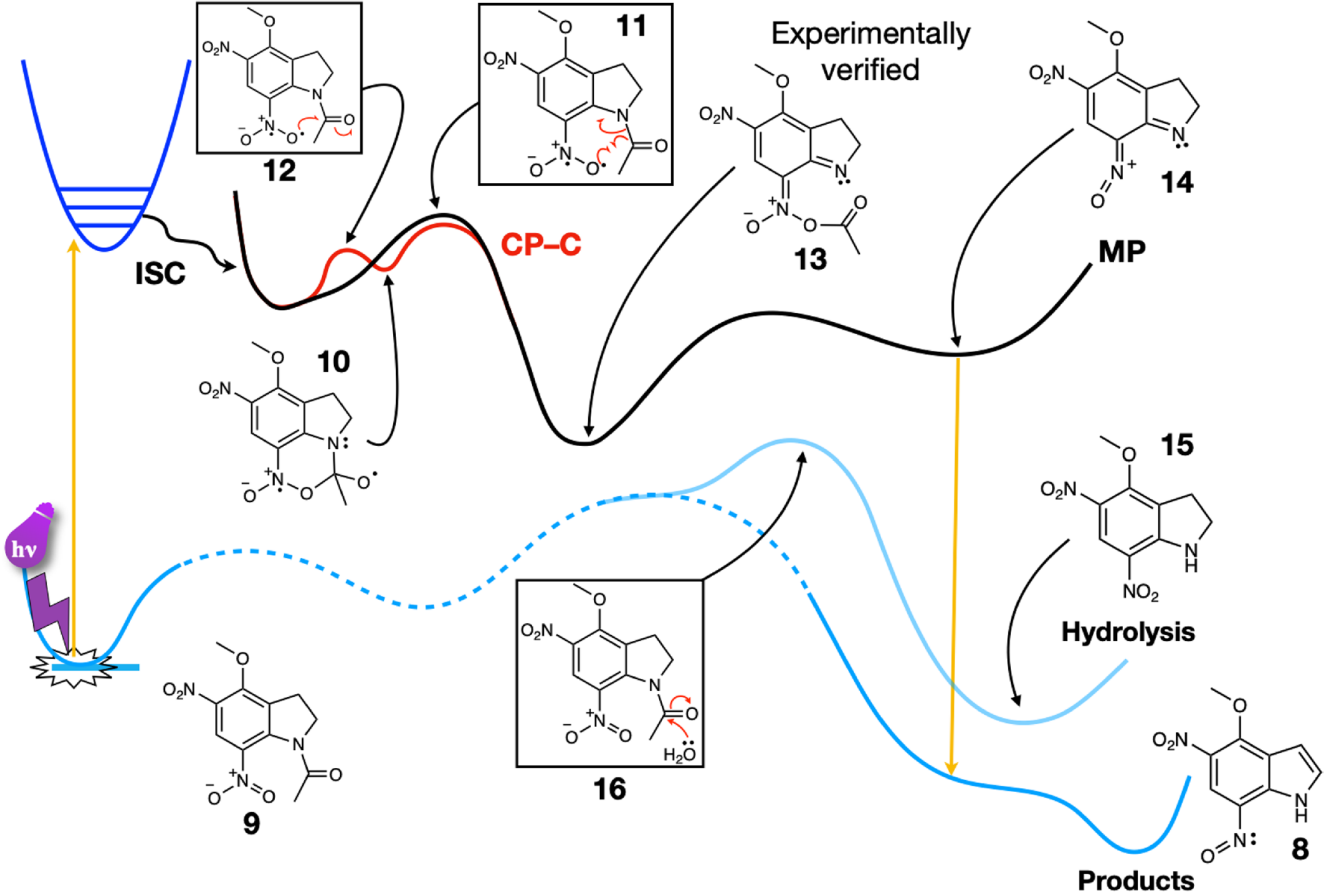
Schematics of the main reaction pathways for MDNI-Ac: Migration Pathway (MP)—mechanism of Pálfi et al., verified by our improved computational results; Cyclization Pathway–C (CP-C) mechanism via cyclic intermediate (see text for a detailed explanation of the pathways). Note that both mechanisms converge towards intermediate **13**. The hydrolysis pathway on the singlet surface is also shown. The structures of the relevant transition states are reported in a box, while significant intermediates and products are reported without a box.

**Figure 3:**
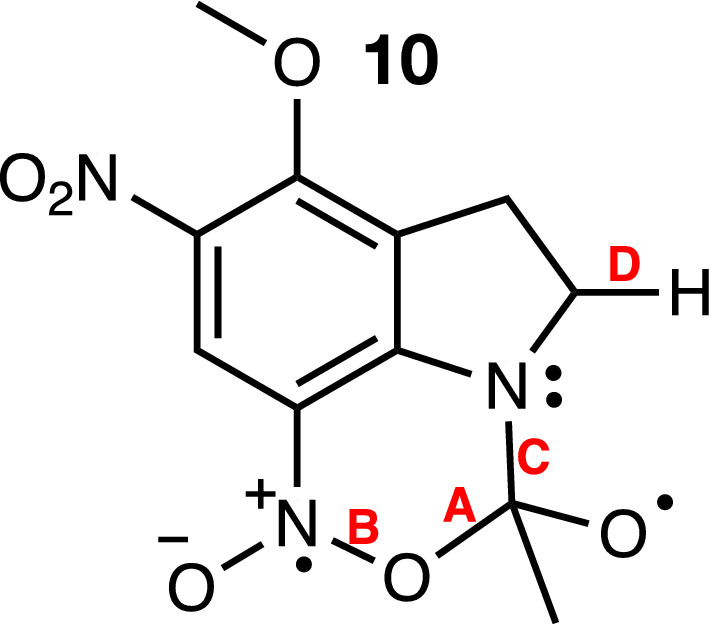
The cyclic intermediate for MDNI-Ac. This structure is similar to structure **5** in Fig. 1, but instead of glutamate, it is bound to acetate.

**Figure 4:**
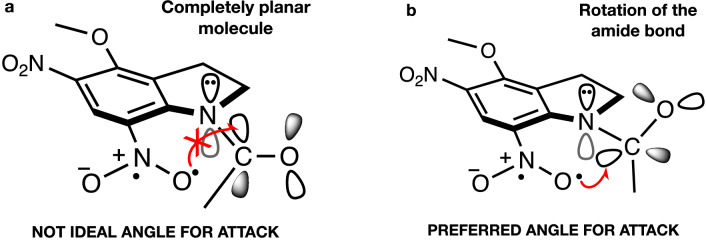
Key electronic effects for the cyclization and migration pathways (CP and MP). The oxygen radical in (**a**) is not positioned at the ideal Bürgi–Dunitz angle of 107° relative to the plane of the molecule, and it is far away from the unoccupied orbital of the carbonyl group. Both pathways necessitate deplanarization of the amide bond via a rotation (**b**) in either sense, leading to two enantiomers (only one shown; see section S2 of the Supplementary Information for more details) in the case of the CP, or directly to the following intermediate in the case of MP.

The reaction pathway from MDNI-Ac (**9** in Fig. 2) to 4-methoxy-5-nitro-7-nitrosoindole (**8**) and acetic acid is thermodynamically favored, with a calculated ΔG of –35.8 kcal/mol (see Fig. 2 below).

According to the CP mechanism, the cyclic intermediate **10** (see below) is the key structure in the reaction mechanisms of MNI and MDNI, regardless of the leaving group. Close inspection of its structure reveals that at least four bonds (labeled A, B, C, or D in Fig. 3) can potentially break, leading to four different routes depending on which of them breaks first. We will label these routes according to the bond that breaks first. Breaking the bond between carbon and oxygen (bond A in Fig. 3) reverts the cyclic intermediate to the reactant, and therefore does not lead to uncaging. Breaking bond B resulted in an unstable intermediate that reverted to the cyclic structure itself during geometry optimization. Coincidentally, it also leads to an intermediate that has not been previously characterized experimentally. For these reasons, we did not explore this pathway any further. The CP mechanism, as proposed initially by Ellis-Davies, involves breaking bond C first, followed by a concerted process initiated by the deprotonation that leads to the reaction products, as seen in Fig. 1. Our calculations show that bond C can indeed be broken, but there is no deprotonation of the resulting intermediate. Instead, once the nitronic anhydride is formed, the leaving group departs, and then deprotonation occurs before the spent cage decays back to the singlet state. Last, we found that the process starting from breaking bond D, followed by the collapse of the cyclic intermediate, is also possible but energetically disfavored compared to the other pathways described above. Breaking bond D, which corresponds to a hydrogen atom abstraction, requires 22.3 kcal/mol for MDNI-Ac.

The MP mechanism involves a migration process that occurs as an alternative to the formation of the cyclic intermediate. According to our results, the migration mechanism is in direct competition with the CP–C cyclization pathway. Both these pathways are consistent with the experimental observations since they both yield the same intermediate—the nitronic anhydride **13** in Fig. 2—identified by Morrison et al. in their kinetic study^40^, and by Cohen et al. in their fast IR spectroscopy study^38^.

The migration pathway necessitates only one step (structure **11** in Fig. 2) to yield the nitronic anhydride **13**, while the cyclization pathway requires two consecutive steps—the formation and the subsequent opening of the cyclic intermediate **10**. The overall energy required to go through these steps is similar, albeit slightly smaller for the cyclization, as seen in Fig. 2. To rationalize this discovery, we looked at a few structural features of the corresponding transition structures (TSs, structures **11** and **12**), as reported in Table 1. Specifically, we considered the bond lengths of bond A and bond C (see Fig. 3), in conjunction with the bond angle they describe. On the one hand, the bond angle is very similar for the two TSs, differing by only 3.7 degrees. This fact shows that the molecule necessitates deplanarization before undergoing any additional change, which exploits the favorable interaction between the available unoccupied orbital of the carbonyl and the singly occupied orbital of the oxygen radical regardless of the pathway (Fig. 4). If the carbonyl group is substituted with an ethyl group, the cyclization pathway is no longer accessible. The migration becomes the only possible pathway, although the electronic effects that make it more accessible are lost (vide infra).

**Table 1:** Calculated bond lengths (in Å) and bond angles (°) in the cyclization and migration transition structures (TSs).

Structure	N–C bond length (Å)	C–O bond length (Å)	N–C–O angle (degrees)
Cyclization TS (**12**)	1.537	1.787	95.4
Cyclic intermediate (**10**, R-Chair)	1.559	1.641	98.4
Migration TS (**11**)	1.709	1.910	91.7

On the other hand, the two bond lengths differ slightly in the two transition structures. They are shorter for the cyclization TS, which justifies why the cyclization process is slightly more favored than the migration process. The acetyl group needs to move farther away from the cage when the migration process occurs, while in the cyclization pathway, the oxygen radical can readily attack the carbon atom without undergoing substantial modifications. The energy requirements for the two reactions are consistent with this concept. The migration process has a slightly larger barrier (5.8 kcal/mol) because the transition structure requires a more significant rearrangement than the cyclization process, which has shorter bonds and a less strict geometric requirement. The cyclization requires 4.7 kcal/mol overall (3.3 kcal/mol for the first step and an additional 1.4 kcal/mol for the ring-opening).

**Figure 5:**
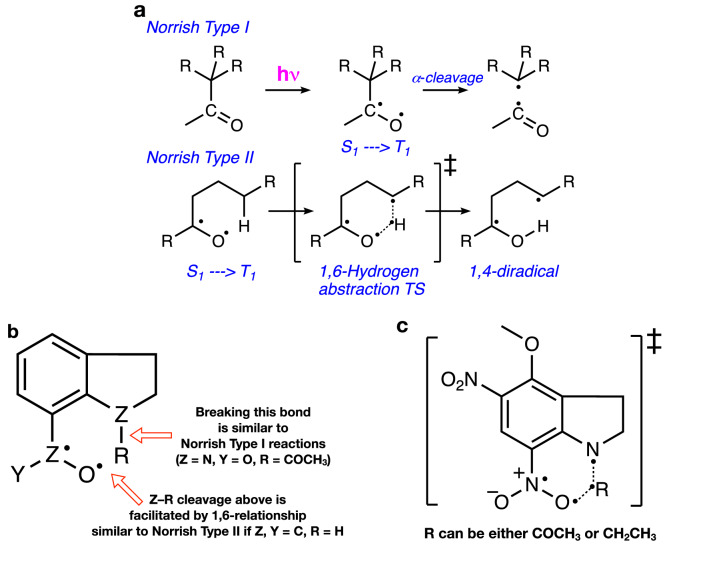
Schematics of the Norrish type reactions (**a**). The MP pathway combines both Norrish type I and type II characteristics (**b**). Breaking the Z–R bond (Z=N, R=COCH_3_) results in a Norrish type I reaction, while the adjacent Z–O bond is in a 1,6-relationship (Norrish type II) and facilitates the cleavage of Z–R. Panel (**c**) shows the key migration transition state. If R=CH_2_CH_3_, the reaction loses the Norrish type I characteristic, and hence, not suprisingly, higher in energy by about 15.5 kcal mol^−1^.

The migration of the acyl unit is facilitated by the inherent formation of the stabilized acyl radical, typically formed within classical Norrish^55^ type I alpha cleavages of excited carbonyl of ketones (Fig. 5a)^56–59^. The 1,6-acyl migration effectively embodies characteristics of both Norrish type I and type II reactions in which the acyl undergoes an α-cleavage as it transfers to the O radical of the nitro unit similar to the γ-H abstraction by triplet carbonyl in Norrish type II positioned at a 1,6-relationship (Fig. 5b)^55,59^. In order to better understand this migration pathway, we computed the energy for an ethyl group migration (in place of the acetyl) and noted that it was higher in energy (21.3 kcal/mol, Fig. 5c), despite being positioned in a 1,6-relationship to the nitro oxygen radical. This reflects the stabilizing effect of an acyl radical in the transition state of the 1,6-migration. The energy difference between the migration and the cyclization pathways is calculated at 1.1 kcal/mol, and since the expected accuracy of our calculations is ± 1.0 kcal/mol^60–64^, we can conclude that the two mechanisms are competitive. As such, given the small activation barriers, both mechanisms can co-occur. We found the same to be true for MNI-Ac as well, with the cyclization requiring 4.8 kcal/mol overall and the migration needing 5.7 kcal/mol.

The inclusion of the entropic contribution to calculate the Gibbs’ free energy profile of the reaction proves that the substitution pattern on 7-nitroindoline is not as important as initially thought. MDNI and MNI require activation energies that are within ~ 2.0 kcal/mol from each other, independently of the side chain of the compound they cage. These similar barriers are evidence for the little influence of the substituents on the crucial steps of the reaction. The energy difference of ∆∆H = 2.0 kcal/mol (calculated with B3LYP/6-31G*, which has an estimated error bar of ~  ± 2.0 kcal/mol) in favor of the MDNI-Glu pathway over the MNI-Glu one reported by Pálfi et al. becomes ~ 4.0 kcal/mol (see Table S3 in the Supplementary Information) when the entropic corrections are appropriately included. In the case of acetate as a caged compound, this difference is almost zero (∆∆G = 0.10 kcal/mol in favor of MNI-Ac for the migration process and ~ 0.10 kcal/mol in favor of MDNI-Ac for the cyclization). All of these values are too small to result in a significant difference in reactivity. In our opinion, the values of the reaction barriers found for both MDNI- and MNI-based cages are also consistent with their current usage as efficient caging agents. Once activated, both structures release the caged compound efficiently, as none of the activation energies are prohibitive. In fact, despite noticeable minimal numerical differences between pathways, the energetic requirements are readily met at room temperature in all cases. Besides, the results of Pálfi et al. show that the largest barrier in the reaction between MNI-Glu and MDNI-Glu is different from the one for α-MNI-Glu and α-MDNI-Glu. The only difference between these two classes of compounds is in the linking point of the cage, as also noted in our previous work in Ref.^37^. In the first case, it is bound to the ω-substituent of the glutamate chain, while in the second case, the cage is bound to the carbon in the α-position, hence the α-M(D)NI notation. Pálfi et al. referred to the two α-structures as MNI-Ulg and MDNI-Ulg, respectively^39^. However, according to their reported data (see Table 1 in Ref.^39^), the enthalpy difference for the highest barrier in the α-case is ∆∆H = 2.3 kcal/mol, a value within the accuracy of the method from the Glu case. For this reason, it is safe to assume that the effect of the substituents on the α-mechanism will be similarly inconsequential, while the origin of the different quantum yields has to be attributed elsewhere. Another significant difference between the results of Pálfi et al. and ours is that we did not find any reaction occurring on the singlet ground state. Pálfi et al. claim that hydrolysis and other side reactions are problematic, as they can readily occur on the singlet ground state (despite a reported ∆H for the hydrolysis reaction barriers of at least 44.6 kcal/mol)^39^. Our new computational results, in conjunction with the experimental findings of Cohen et al.^38^, contradict the hypothesis of other side reactions occurring on the singlet surface. It is noteworthy that if hydrolysis occurred, then the cage would lead to the uncontrolled release of the active compound, invalidating its application as a reliable cage, and additionally would have been experimentally observed by Cohen et al. and in our laboratory. Furthermore, the hydrolysis product (**15**) and the uncaging product (**8**) are different in that the former retains the nitro group while the photo uncaging leads to the nitroso. However, both processes result in the release of acetic acid (from **9** and **13,** respectively).

**Figure 6:**
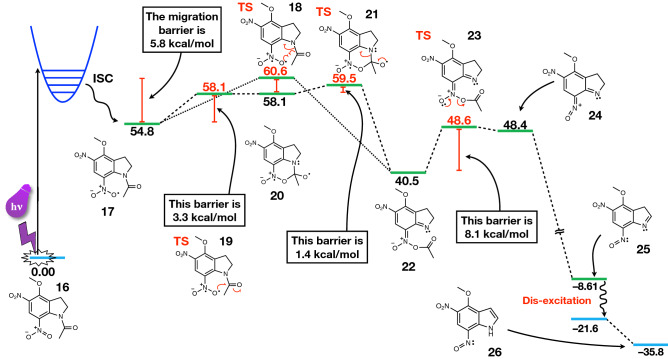
The reaction mechanism for the uncaging of MDNI-Ac. The singlet surface is indicated in blue, while the triplet surface is indicated in green. The CP–C mechanism is outlined with the dashed line, while the MP mechanism follows the dotted line. They both converge towards structure **22**, and then they proceed towards the products in the same way. The reported numerical values are Gibbs free energies calculated with respect to MDNI-Ac in the singlet state (structure **16**), and they have been obtained at the ωB97X-D/def2-TZVP level of theory in water (C-PCM). All values are in kcal/mol. Structures **16**, **18**, **19**, **20**, **22**, **24,** and **26** have been renumbered for better clarity, and they correspond to structures **9**, **11**, **12**, **10**, **13**, **14,** and **8**, respectively, in Fig. 2.

In summary, our improved computational protocol on the uncaging mechanism shows that the lowest energy path proceeds as follows. After irradiation, the first step is either the migration of the acetyl group from the nitrogen atom of indoline to one of the oxygen atoms of the neighboring nitro group (dotted line in Fig. 6) or the formation of the cyclic intermediate and its subsequent opening (dashed line in Fig. 6). The migration transition structure (structure **18**) is only 5.8 kcal/mol higher than the reactant, and it is accessible at room temperature. The cyclization step involves two subsequent reactions having an activation barrier of 3.3 and 1.4 kcal/mol, respectively (structures **19** and **21** via intermediate **20** in Fig. 6). In all cases, the energy barriers are non-prohibitive, making both routes plausible at room temperature. Both pathways converge towards the formation of the nitronic anhydride (structure **22**). The subsequent step (**23**) has a barrier of 8.1 kcal/mol. After the leaving group (acetate in this case) is released (**23**), a charged nitroso intermediate then forms (**24**), which readily deprotonates forming **25**, which then dis-excites, going back to the singlet surface. This is also consistent with the fact that molecules including an aromatic ring are more acidic in the excited state than in the ground state^65–68^. Subsequently, the non-aromatic five-membered ring in **25** spontaneously tautomerizes to the fully aromatic indole system of the spent cage, 4-methoxy-5-nitro-7-nitrosoindole (**26**). The overall reaction mechanism for MDNI-Ac is outlined in Fig. 6.

**Figure 7:**
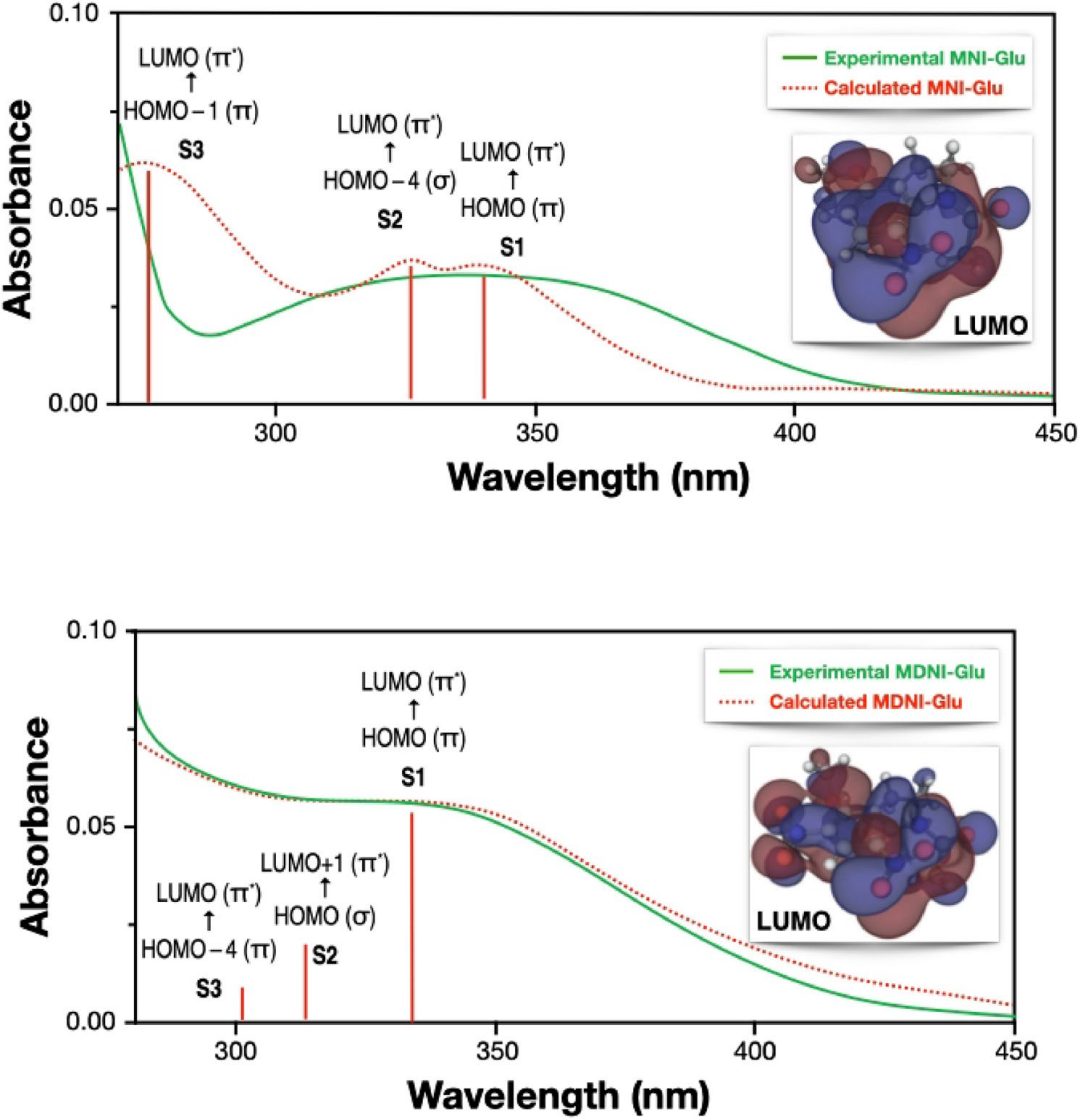
Comparison of the experimental (green, solid) and calculated (red, dashed) UV–Vis spectra of (**a**) MNI (top panel) and (**b**) MDNI (bottom panel). The calculated spectra are obtained by applying a Lorentzian broadening to the ωB97X-D/def2-TZVP electronic transitions, also reported as red vertical lines. The canonical π^*^ lowest unoccupied molecular orbitals involved in the bright electronic transitions are also reported. The experimental spectra have been obtained in water (pH = 7.39 with NaHCO_3_), at a concentration of 0.074 mM and using radiation at 350 nm as explained in Ref.^36^.

Having excluded the uncaging mechanism on the triplet surface as a potential explanation of the quantum yield, the most likely steps that can affect the behavior of this family of molecules are the response towards the incident light and how readily the triplet state is populated. The *true* reactant is the molecule in the triplet state. Motivated in a slight redshift observed experimentally in the UV–Vis absorption spectrum going from MNI-Glu to MDNI-Glu, we contend that controlling the amount of reactant in the triplet state is the key to achieving higher quantum yields. Such control can be achieved by introducing different substituents on the cage moiety. To computationally characterize the excited states of the molecules, we performed time-dependent density functional theory calculations with the Tamm–Dancoff approximation^69^ using several exchange–correlation functionals, and we compared them with the experimental UV–Vis spectra of MNI-Ac and MDNI-Ac. The range-separated ωB97X-D showed the best agreement with the experimental spectra, as shown in Fig. 7, and therefore it was chosen as the reference exchange–correlation functional for the entire study.

A singlet–triplet transition is strictly forbidden in non-relativistic quantum mechanics, but it can happen, in practice, because of intersystem crossing (ISC). Higher reaction quantum yields generally correspond to higher rates of ISC, as established, for example, for the boron-dipyrromethene (BODIPY) family of cages^23,70^. Both MDNI and MNI have three singlet–singlet transitions in the relevant region of the UV–Vis spectrum (250–500 nm, Fig. 7), but it is sufficient to focus on the first singlet excited state (S_1_), as the internal conversion between any higher singlet state and the S_1_ state is usually faster than any other process^71^. Our TD-DFT results reported in Table 2 show that MDNI-Glu has five triplet states below the first singlet, while MNI-Glu has only three. According to El-Sayed’s rule^72^, the rate of ISC is larger for transitions that involve a change of molecular orbital type. The orbital analysis of the relevant triplet states of MNI- and MDNI-Glu (Table 2) reveals that the latter has two electronic transitions presenting this characteristic (S_1_ → T_4_ and S_1_ → T_5_, which go from a ^1^(π → π*) state to a ^3^(σ → π*) state), while the former has only one (the (S_1_ → T_3_), which is a ^3^(σ → π*) transition). These results qualitatively explain the faster ISC for MDNI compared to MNI since it has access to twice as many triplet states. More details on the analysis of the transitions between the S_1_ state and the triplet states are also reported in Table S5 in the Supplementary Information. The introduction of better chromophores represents the key to achieving better quantum yields since modifications of the electronic structure of the cages can be achieved by changing the substituents on their scaffolds.

**Table 2:** Details of the excitation types for the singlet and triplet states in the experimental range (250–500 nm) together with the excitation energy (in eV) for MNI-Glu and MDNI-Glu.

Molecule	State	Energy (eV)	Orbital Character	Molecule	State	Energy (eV)	Transition Character
MNI-Glu	S_1_	3.62	^1^(π → π*)	MDNI-Glu	S_1_	3.64	^1^(π → π*)
	S_2_	3.93	^1^(σ → π*)		S_2_	3.91	^1^(σ → π*)
	S_3_	4.35	^1^(π → π*)		S_3_	4.00	^1^(σ → π*)
	T_1_	3.01	^3^(π → π*)		T_1_	2.96	^3^(π → π*)
	T_2_	3.18	^3^(π → π*)		T_2_	3.30	^3^(π → π*)
	T_3_	3.54	^3^(σ → π*)		T_3_	3.42	^3^(π → π*)
	T_4_	3.66	^3^(σ → π*)		T_4_	3.47	^3^(σ → π*)
	T_5_	4.02	^3^(π → π*)		T_5_	3.48	^3^(σ → π*)
	T_6_	4.20	^3^(σ → π*)		T_6_	3.69	^3^(σ → π*)
	N/A				T_7_	3.75	^3^(σ → π*)

## Conclusion

In conclusion, we present a detailed description of the reaction mechanism of the photouncaging of 4-methoxy-5,7-dinitroindolinyl acetate (MDNI-Ac) and 4-methoxy-7-nitroindolinyl acetate (MNI-Ac). We show that the mechanism proceeds in the triplet state through two competitive pathways via the formation of a cyclic intermediate or through a combined Norrish type I and type II 1,6-acyl migration to the adjacent nitro group. We analyze the geometric and electronic features of the competitive routes. Both pathways yield a nitronic anhydride, which subsequently releases the leaving group of the nitronic ester, while the spent cage is recovered in the singlet ground state. The reason behind the superior photoproperties of MDNI as a better caging group does not lie in lower activation energies but rather in a better sensitivity towards light due to an increased intersystem crossing rate. This is possible because the second nitro group on the MDNI cages lowers the energy of the triplet states with respect to the first singlet state, thus increasing their availability to undergo ISC. Our findings provide additional insights into the reactivity of the MDNI family of photocages and guidance for the future synthesis of improved cage designs.

## Methods.

### Computational methods and models

Details about the computational methods, the theoretical frameworks, and the computational programs used can be found in Section S13 in the Supplementary Information. The appropriate references are detailed in the Reference section (Section S15) of the Supplementary Information. Last, sample input files for all calculations are also provided (Section S14 in the Supplementary Information).

## Supplementary Information

Supplementary Information 1.

Supplementary Information 2.

Supplementary Information 3.

Supplementary Information 4.
