# Supplementary material for: Competition between cyclization and unusual Norrish type I and type II nitro-acyl migration pathways in the photouncaging of 1-acyl-7-nitroindoline revealed by computations
Source: Sci Rep. 2021 Jan 14;11:1396. doi: 10.1038/s41598-020-79701-4 (PMC7809399; doi:10.1038/s41598-020-79701-4)
Supplement: Supplementary file 1 — Supplementary Information 1. [file 41598_2020_79701_MOESM1_ESM.docx]

**Supplementary Information for**

**Competition Between Cyclization and Unusual Norrish Type I and Type II Nitro-Acyl Migration Pathways in the Photouncaging of 1-Acyl-7-nitroindoline** **Revealed by Computations**

Pierpaolo Morgante, Charitha Guruge, Yannick P. Ouedraogo,

Nasri Nesnas*, Roberto Peverati*

Chemistry Program, Florida Institute of Technology,

150 W. University Blvd. 32901, Melbourne, FL, United States

E-mail: [nesnas@fit.edu](mailto:nesnas@fit.edu); [rpeverati@fit.edu](mailto:rpeverati@fit.edu)

Section S1, page S1: Organization of the Geometries Folder.

Section S2, page S2: Comparing Different Exchange-Correlation Functional Approximations for the Reaction’s Mechanisms.

Section S3, pages S2–S5: The stereochemical features of the cyclic intermediate for MDNI-Ac and MNI-Ac.

Section S4, pages S5–S6: Multi-reference Character of the Relevant Transition Structures of the Main Mechanism for MDNI-Ac.

Section S5, pages S6–S8: Reaction Mechanisms of MDNI-Ac Involving Cyclization.

The Cyclization Pathway–D (CP–D).

Section S6, pages S8–S9: Reaction Mechanisms of MDNI-Ac Involving Cyclization. The Cyclization Pathway CP–B and Singlet Reactivity.

Section S7, pages S10–S11: Reaction Mechanism for MNI-Ac.

Section S8, pages S11–S12: Multi-reference Character of the Relevant Transition Structures of the Main Mechanism for MNI-Ac.

Section S9, pages S12–S13: Reaction Mechanisms of MNI-Ac Involving Cyclization. The Cyclization Pathway–D (CP–D).

Section S10, pages S14–S15: Reaction Mechanisms of MNI-Ac Involving Cyclization. Pathway CP–B and Singlet Reactivity.

Section S11, pages S15–S16: Compounds containing glutamate.

Section S12, pages S17–S23: More details on the TD-DFT calculations.

Section S13, pages S23–S25: Computational Details.

Section S14, pages S26–S27: Detailed Description of the Procedure Used to Correct the Structures with More than One Negative Eigenvalue.

Section S15, pages S27–S28: Sample Input Files.

Section S16, pages S29–S33: References

**Section S1: Organization of the Geometries Folder.**

The attached Geometries folder is organized in four sub-folders named Acetate, Glutamate, MDNI-Et, and TD-DFT.

In the Acetate folder, we collected all the structures belonging to the pathways described in the main text and here for MDNI-Ac and MNI-Ac. We named them according to the structure’s number in the main text or the Supplementary Information. For example, MDNI-Ac in the singlet state (structure **16** from **Figure 6** of the main text, and also **S1** in **Supplementary Figures S3**) has been named Structure16_S1.xyz. All the other structures have been named following this scheme. A key is also provided in the Excel file where all the calculated electronic and Gibbs free energy values have been reported. The geometries used for the TD-DFT calculations are instead collected in the folder called TD-DFT. They are named according to the compound’s name. The geometries used for MDNI-Et are collected in the MDNI-Et folder. As for the TD-DFT structures, they are named according to the compound’s name. The geometries for caged glutamate, instead, are collected in sub-folders depending on the how the compound is bonded to the cage.

The calculated electronic and Gibbs free energies are reported in the Excel files, together with the numerical results for the multi-reference diagnostics, and the quantum yield calculations.

**Section S2: Comparing Different Exchange-Correlation Functional Approximations for the Reaction’s Mechanisms.**

As pointed out in the main text, we performed our computational study with many exchange-correlation functional approximations. We included the range-separated hybrid Generalized Gradient Approximation (GGA) functionals LC-ωHPBE,^1^ and CAM-B3LYP^2^ and ωB97X-D,^3^ and the range-separated hybrid meta-GGAs M11^4^ and ωB97M-V.^5^ Also, we tested the hybrid meta-GGA functional MN15,^6^ and the dispersion-corrected double-hybrid DSD-PBEP86-D3(BJ).^7–9^ These functionals, especially the Minnesota- and ωB97-families, are known to work well for both ground-state^6,10,11^ and excited-state properties.^12–15^ Our preference for ωB97X-D was dictated by the excellent agreement between the experimental UV-VIS spectrum with the one calculated at this level of theory (**Figure 7** in the main text). To give a comprehensive theoretical description of the reaction, we then decided to adopt this functional for the Gibbs free energy results presented in the main text and the following **Sections S3–S11**. All the functionals we tested performed similarly, yielding the same description of the reaction pathways analyzed in this study. We refer to the Excel files for the detailed numerical results. All the functionals depict the same reaction mechanism, with only minor changes with respect to the ωB97X-D results that we present here and in the main text. The same conclusions can be drawn by looking at the results obtained with any of the functionals reported above.

**Section S3: The stereochemical features of the cyclic intermediate for MDNI-Ac and MNI-Ac.**

The cyclic intermediate is the key structure in the mechanism proposed by Ellis-Davies^16^ and Morrison et. al^17^ (Cyclization Pathway, CP, *vide infra*). We also found out that the cyclization mechanism is the lowest-lying pathway for the reaction. Therefore, understanding the features of the cyclic intermediate is critical for the correct interpretation of the results obtained in this study. A new stereocenter is formed when either MNI- or MDNI-Ac react to form the six-membered cyclic intermediate, and its configuration (R or S) depends on the geometry of attack. At the same time, the orientation of the attack can also lead to two different conformations of the ring, which can be either chair- or boat-like (it is not properly a chair or a boat because the aromatic ring is planar). In total we have a combination of four structures, which come from either configuration of the stereocenter with either conformation of the ring. Namely, we have R-Chair, R-Boat, S-Chair and S-Boat (all reported in **Supplementary** **Figure S1**, structures **A** through **D**), whose acronyms have been chosen as a quick way to identify the configuration and the conformation of the structure.

We might expect that the two structures labeled R-Boat and S-Chair are more stable than the others because of the position of the methyl group. In fact, in these two cases it lies outside the rest of the ring, in an *exo* position, while in the two conformers R-Chair and S-Boat the methyl group is in an *endo* position. Surprisingly, all four intermediates have almost the same energy, as indicated in **Supplementary** **Figure S1** by the relative differences in Gibbs free energy (∆∆G). The position of the methyl substituent therefore is not as important as we first expected. Since the energy difference we found is below the accuracy of the method we used, we can conclude that the structures are practically equivalent. Since the cyclic intermediates have all the same energy, we focused only on the R-Chair structure (**Supplementary** **Figure S1,** structure C).

| **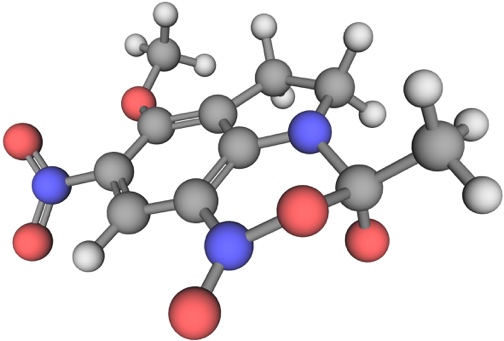** | **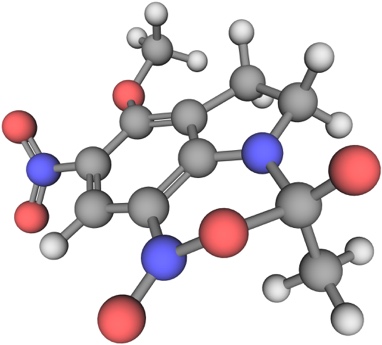** |
| --- | --- |
| **** | **** |
| **A) R-Boat, ∆∆G=0.00 kcal/mol** | **B) S-Boat, ∆∆G=2.09 kcal/mol** |
| **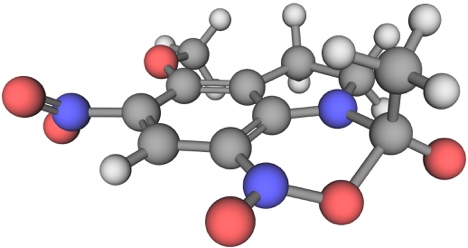** | **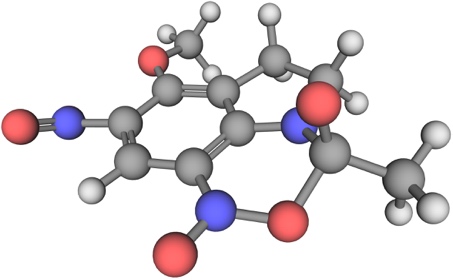** |
| **** | **** |
| **C) R-Chair, ∆∆G=1.16 kcal/mol** | **D) S-Chair, ∆∆G=0.00 kcal/mol** |
| **Supplementary Figure S1** The four cyclic structures taken into account based on the configuration of the stereocenter (R or S) and the conformation (boat or chair) of the cyclohexene ring for MDNI-Ac. The carbon atoms are in black, hydrogens in white, nitrogens in blue and oxygens in red. Skeletal formulas (hydrogen atoms are omitted) are included for clarity. | |

The consideration we made for the cyclic intermediate of MDNI-Ac (**Supplementary** **Figure S1**) can be applied to the MNI-Ac case as well. In fact, we analyzed the structure of the four cyclic intermediates resulting from cyclization of MNI-Ac (**Supplementary** **Figure S2**) and we assigned their names in a manner analogous to the cyclic structures of MDNI-Ac. We found that their relative energies lie within 2.0 kcal/mol from each other. In this case as well, we can safely assume that the difference in energy among the intermediates is almost the same, and there is no difference in their energetics.

| **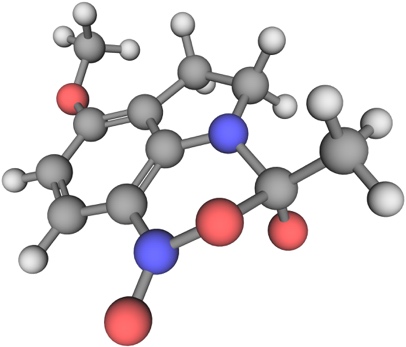** | **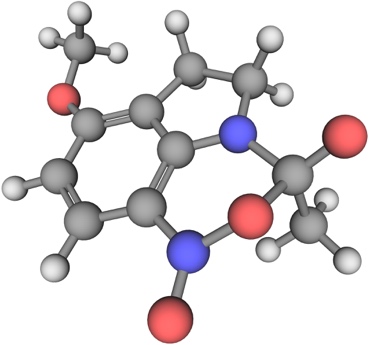** |
| --- | --- |
| **** | **** |
| **A) R-Boat, ∆∆G=0.00 kcal/mol** | **B) S-Boat, ∆∆G=1.99 kcal/mol** |
| **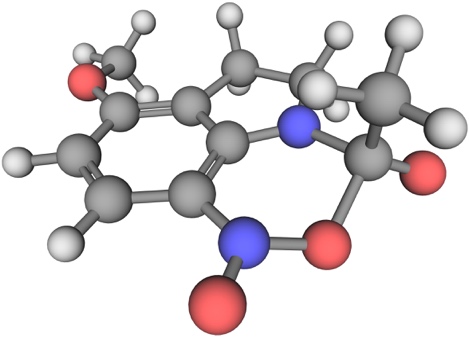** | **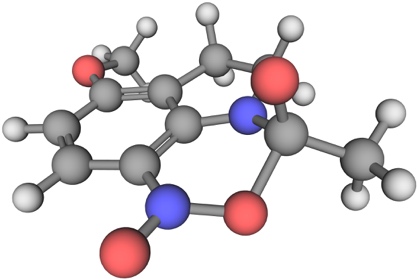** |
| **** | **** |
| **C) R-Chair, ∆∆G=1.12 kcal/mol** | **D) S-Chair, ∆∆G=0.00 kcal/mol** |
| **Supplementary** **Figure S2** The four cyclic structures taken into account based on the configuration of the stereocenter (R or S) and the conformation (boat or chair) of the cyclohexene ring for MNI-Ac. The carbon atoms are in black, hydrogens in white, nitrogens in blue and oxygens in red. Skeletal formulas (hydrogen atoms are omitted) are included for clarity. | |

**Section S4: Multi-reference Character of the Relevant Transition Structures of the Main Mechanism for MDNI-Ac.**

To make sure that density functional theory (DFT) is indeed capable of providing an accurate answer for this reaction mechanism, we assessed the multi-reference (MR) character of the relevant transition structures for the MDNI-Ac migration and cyclization pathways. Namely, we focused on the cyclization, migration and leaving group departure steps. For the B_1_ diagnostic^18^ of Schultz, Zhao and Truhlar we computed the bond dissociation energy of the reaction from structure **17** to structure **22**, from structure **17** to structure **20**, from structure **20** to structure **22**, and from structure **22** to structure **24** of the main text (refer to **Figure 6** for the structures’ numbers; more details on the procedure can be found in **Section S13**). The numerical values are reported in **Table S1** below, and in the Excel file “results_MDNI.xlsx”. All of them are below the threshold of 10.0 kcal/mol indicated by Schultz, Zhao and Truhlar.^18^ Therefore, all steps are unproblematic cases.

This result has been cross validated with the A_λ_ diagnostic^19^ of Martin and coworkers (more details on the procedure can be found in **Section S13**). In this case, we directly applied it to the transition structures (TSs) **18**, **19**, **21** and **23**, with the numerical values reported in the table below. All cases fall within the range of unproblematic values, and we concluded that DFT is reliable for the description of this reaction mechanism.

| **Table S1** B_1_ and A_λ_ diagnostics on relevant transition structures and steps of the migration and cyclization pathways for MDNI-Ac. The structure numbers refer to **Figure 6** of the main text. | |
| --- | --- |
| **Structure^a^** | **A_λ_ diagnostics** |
| Migration TS (**18**) | 0.154 |
| Cyclization TS (**19)** | 0.151 |
| Ring-opening TS (**21**) | 0.150 |
| Leaving group departure TS (**23**) | 0.153 |
| **Structure^a^** | **B_1_ diagnostics^b^** |
| Formation of **22** from **17** through **18** | 8.26 |
| Formation of **20** (R-Chair) from **17** | 1.91 |
| Formation of **22** from **20** | 6.35 |
| Formation of **24** from **22** | 9.43 |
| ^a^ Refer to **Figure 6** of the main text to identify the structures from their numbers.  ^b^ units are kcal/mol | |

**Section S5: Reaction Mechanisms of MDNI-Ac Involving Cyclization.**

**The Cyclization Pathway–D (CP–D).**

In 2005,^16^ Ellis-Davies proposed a mechanism for the uncaging of 4-methoxy-5,7-dinitroindolinyl glutamate (MDNI-Glu) following a previously reported pathway for the decay of the methyl ester of 4-carboxymethoxy-7-nitroindolinyl acetate.^17^ We named this mechanism Cyclization Pathway (CP, **Figure 1** of the main text). CP involves the formation of the cyclic intermediate, and then its subsequent collapse via a concerted process that involves deprotonation and the departure of the leaving group. We found that this concerted mechanism is actually split into two separate steps, as reported in **Figure 6** of the main text, and proceeds through the formation of the cyclic intermediate, its breakdown, and the subsequent deprotonation. As noted in the main text, we labeled the different pathways according to the bond that breaks first in that pathway. We reported the cyclization pathway–C (CP–C) in **Figure 6** of the main text, together with the migration pathway (MP).

The other mechanism that is consistent with Ellis-Davies and Morrison et al. idea is reported in **Supplementary** **Figure S3**, and we label it CP–D. The reason behind this name is because it starts with the breakage of bond D (see structure **9** in the main text). In this case, MDNI-Ac cyclizes (structures **S2–S4** in **Supplementary Figure S3**), and then it undergoes a hydrogen atom abstraction (**S5**). This step yields the formation of a “protonated” intermediate (**S6**), which then collapses in an almost concerted way to yield acetate and the cage. It makes sense to represent this step as concerted for two reasons. First, the second transition structure (**S7**) is lower in energy than the first (**S8**); second, there is no intermediate between them, and the two transition structures are subsequent to each other. The cage then undergoes decay to the singlet surface (**S9**) in an analogous manner to CP–C and MP, and then it tautomerizes (**S10**). Given the high barrier (22.3 kcal/mol), this pathway is less likely to happen than those reported in the main text.

| 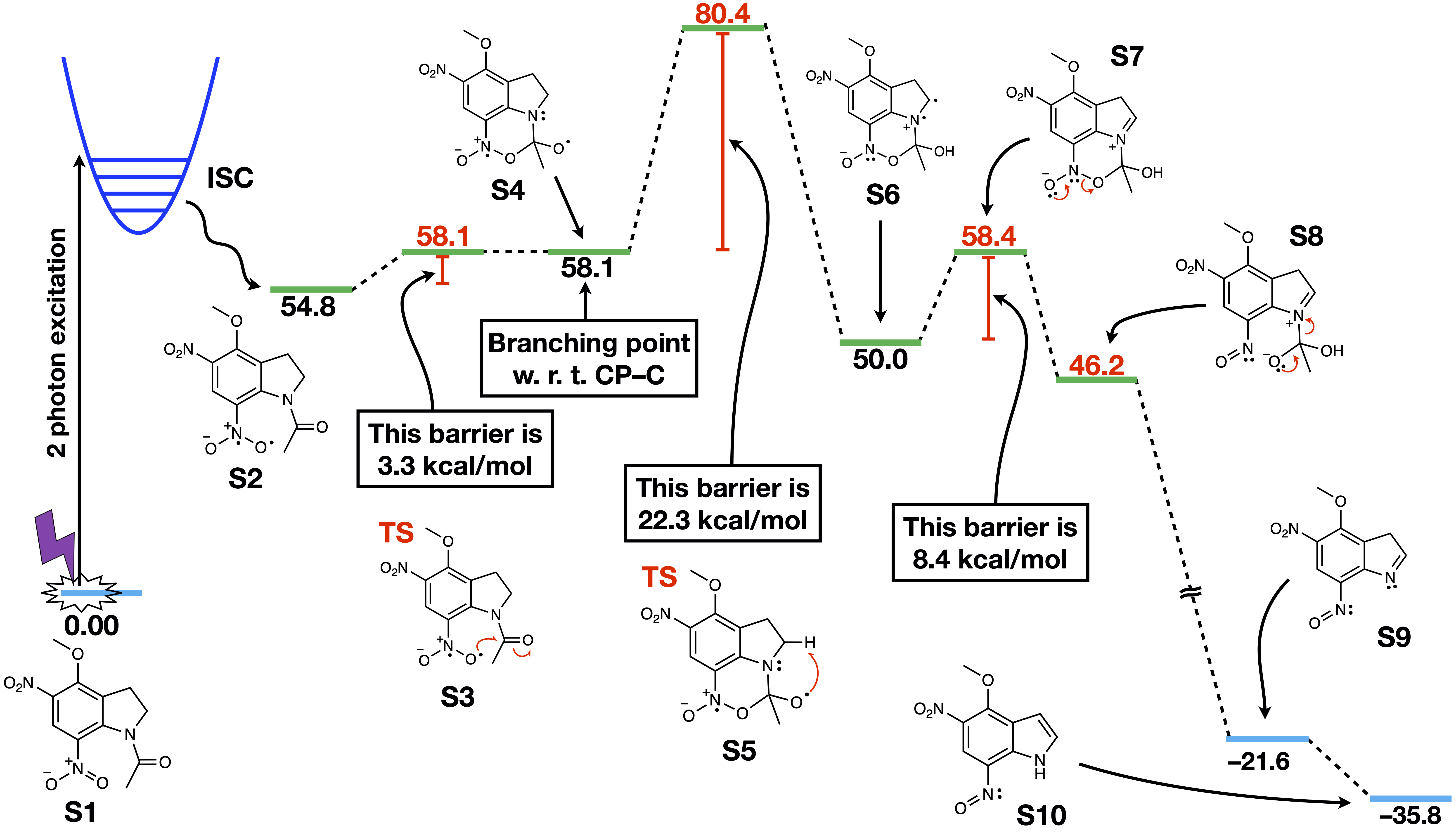 |
| --- |
| **Supplementary Figure S3** The CP–D reaction mechanism for MDNI-Ac. The triplet surface is reported in green, while the singlet surface is in blue. The reported numerical values are Gibbs free energies calculated with respect to MDNI-Ac in the singlet state (structure **S1**), and they have been obtained at the ωB97X-D/def2-TZVP level of theory in water (C-PCM). All values are in kcal/mol. |

In summary, Ellis-Davies and Morrison et al. idea (CP) is consistent with two mechanisms. However, the pathway they indicated (**Figure 1** in the main text) includes steps that are proper of two different mechanisms, as shown by **Figure 6** of the main text (CP–C) and **Supplementary Figure S3** (CP–D). It is worth noticing that while in the CP–C pathway, there is a deprotonation step, in the CP–D pathway, there is a hydrogen atom abstraction instead.

**Section S6: Reaction Mechanisms of MDNI-Ac Involving Cyclization. The Cyclization Pathway CP–B and Singlet Reactivity.**

We grouped here the last two side-mechanisms that we explored. The first is another “branch” of the pathway, starting from the cyclic intermediate on the triplet surface. Since it involves breakage of bond B of structure **9** in the main text, we call this mechanism CP–B. The formation of **S4** is the same that we described before (**Figure 6** of the main text, structures **17**, **19** and **20**, and **Supplementary Figures S3**), but in this case, it evolves by breaking the N–O bond (structure **S11** in **Supplementary Figure S4**). The activation energy needed for this process is 22.2 kcal/mol, which is as high as the hydrogen abstraction described in **Supplementary Figure** **S3**. However, we could not locate any other structure for the intermediate immediately following this transition state, despite multiple attempts with different search algorithms. During geometry optimization, the structure kept reverting to the cyclic structure **S4**. Due to the prohibitive reaction barrier, this pathway is not likely to occur, and due to the fact that it would not lead to an experimentally characterized intermediate, we did not explore it any further.

| 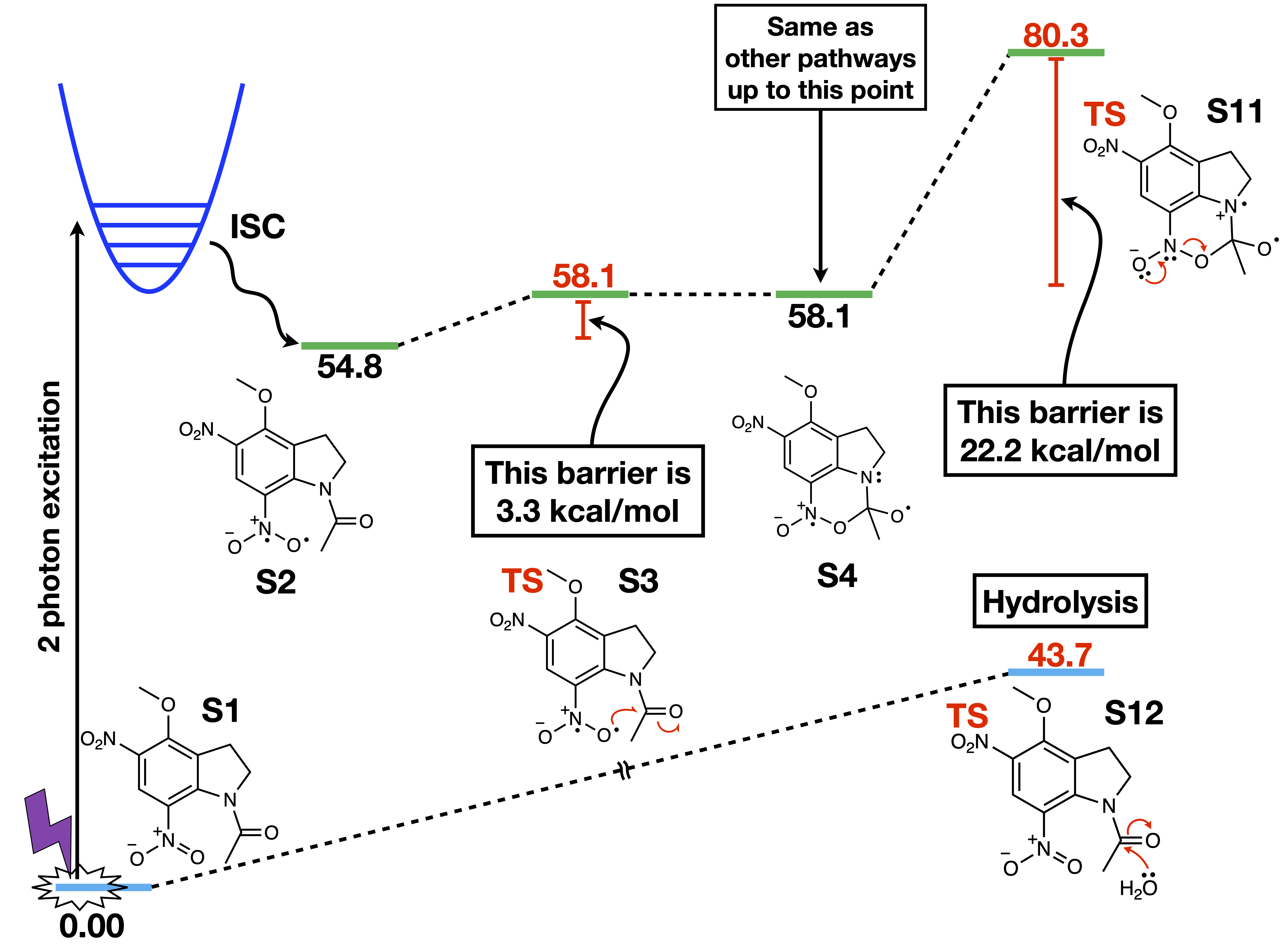 |
| --- |
| **Supplementary Figure S4** The CP–B mechanism (upper section) and the hydrolysis mechanism (lower section) for MDNI-Ac. The triplet surface is reported in green, while the singlet surface is in blue. The reported numerical values are Gibbs free energies calculated with respect to MDNI-Ac in the singlet state (structure **S1**), and they have been obtained at the ωB97X-D/def2-TZVP level of theory in water (C-PCM). All values are in kcal/mol. |

We located two interesting transition structures on the singlet surface as well. We wanted to make sure that the cyclization process would not occur on the singlet surface. We explored the potential energy surface, and we located the cyclization transition structure (analogous to structure **19** in **Figure 6** of the main text) at 35.1 kcal/mol above the reactant. This is enough to ensure that there is no reaction on the singlet surface.

The second transition structure we wanted to locate is related to the hydrolysis process, as according to Pálfi et al. it represents a problem for MDNI- or MNI-Glu.^20^ The activation energy of this process is 43.7 kcal/mol (the hydrolysis transition structure is structure **S12** in **Supplementary Figure S4**). We do not believe hydrolysis represents an important side-process, as it has not been observed experimentally.^21^ Our calculations confirm that it requires too much energy to occur in any appreciable way.

**Section S7: Reaction Mechanism for MNI-Ac.**

The reaction mechanism reported in **Supplementary Figure S5** below can be directly compared to the reaction mechanism in **Figure 6** of the main text. For MNI-Ac, we see that the reaction happens in the triplet surface, as for MDNI-Ac. We observe a competition between the migration and cyclization pathways as we did for MDNI-Ac. The first step is either the acyl transfer between the nitrogen atom of the indoline scaffold (**S15** in **Supplementary Figure S5**) and one of oxygen atoms of the nitro group, or the cyclization process. The migration requires 5.7 kcal/mol (it requires 5.8 kcal/mol for MDNI-Ac, structure **18** in **Figure 6**), while the cyclization transition structure (**S16**) requires only 2.4 kcal/mol, thus representing the lowest energy pathways. The cyclic structure **S17** then open up yielding the nitronic anhydride **S21** after overcoming a barrier of 2.5 kcal/mol. Overall, the energy required for the cyclization is 4.9 kcal/mol, only 0.8 kcal/mol less than the migration pathway. For MNI-Ac as well, the migration and cyclization processes are equivalent, and they yield the same intermediate (**S17**). Once the intermediate **S17** is formed, the leaving group departs after a barrier of 7.9 kcal/mol is overcome (**S22**). This step requires slightly less energy than MDNI-Ac (which needs 8.1 kcal/mol, structure **23** in **Figure 6**). The charged intermediate **S21** then deprotonates and goes back to the singlet surface (**S22**) and it yields the spent cage **S23** after tautomerization. The Gibbs free energy of the reaction amounts to –30.6 kcal/mol. Overall, MNI-Ac and MDNI-Ac react in a very similar way, and the differences in the mechanisms are negligible.

| 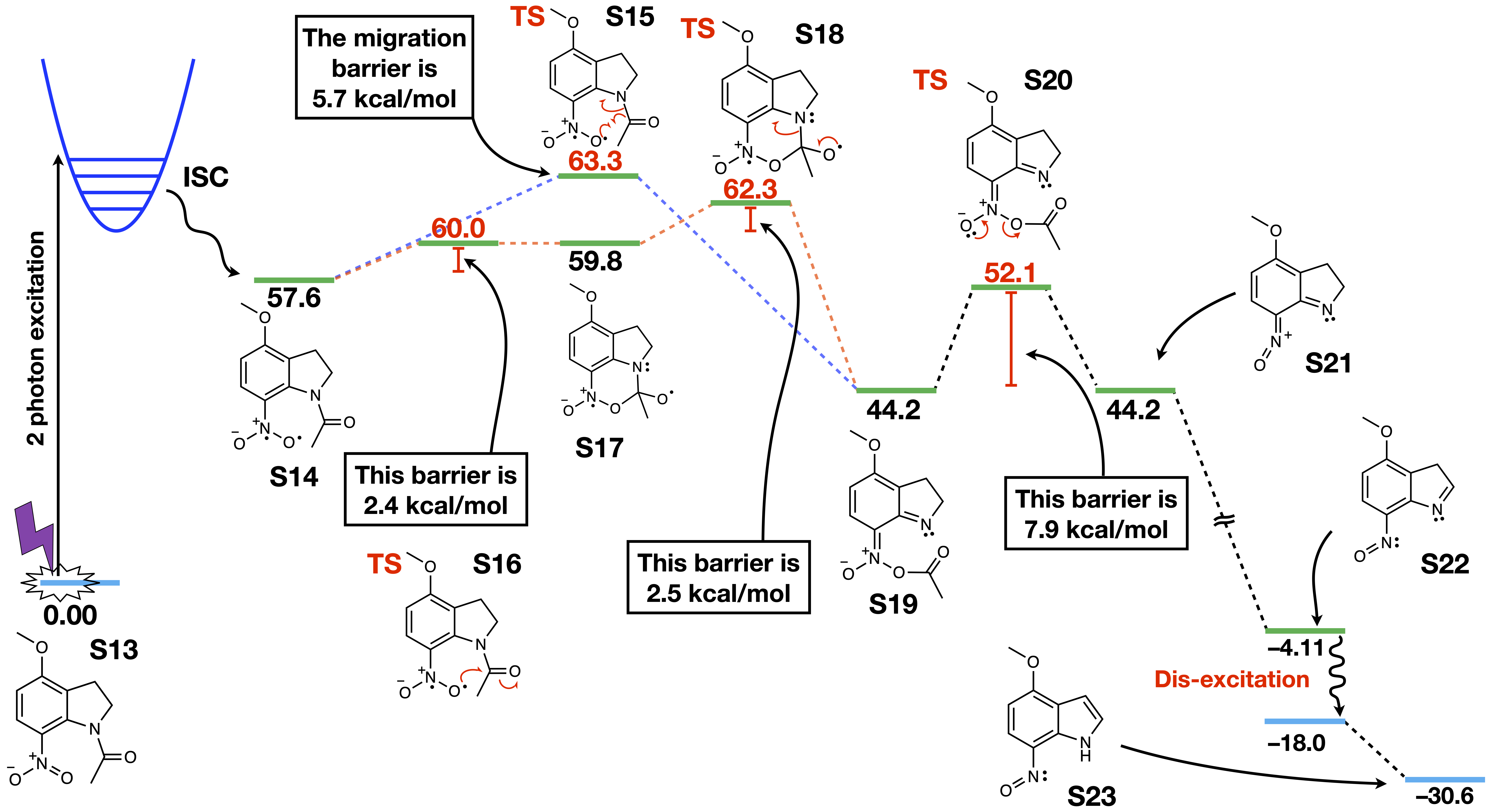 |
| --- |
| **Supplementary Figure S5** The reaction mechanism for MNI-Ac including the CP–C pathway (dotted orange line) and the MP (dotted blue line). CP–C and MP converge towards the same nitronic anhydride **S19**. The triplet surface is reported in green, while the singlet surface is in blue. The reported numerical values are Gibbs free energies calculated with respect to MNI-Ac in the singlet state (structure **S13**) and they have been obtained at the ωB97X-D/def2-TZVP level of theory in water (C-PCM). All values are in kcal/mol. |

**Section S8: Multi-reference Character of the Relevant Transition Structures of the Main Mechanism for MNI-Ac.**

As we did for MDNI-Ac, we tested the reliability of the single-determinant treatment for the main intermediates and transition structures in the main mechanism of MNI-Ac as well. We analyzed the same steps we tested for MDNI-Ac (the cyclization, migration and leaving group departure steps). For the B_1_ diagnostic^18^ of Schultz, Zhao and Truhlar (more details on the procedure can be found in **Section S13**) we computed the bond dissociation energy of the reaction from structure **S14** to structure **S19**, from structure **S14** to **S17**, from structure **S17** to structure **S19**, and from structure **S19** to **S21**. The values we obtained are reported in **Table S2** below. All cases are below the threshold of 10.0 kcal/mol indicated by Schultz, Zhao and Truhlar to characterize unproblematic cases.^18^

These results have been cross validated with the A_λ_ diagnostic^19^ of Martin and coworkers (more details on the procedure can be found in **Section S13**). In this case, we directly applied it to the transition structures **S15**, **S16**, **S18**, and **S20**. The values we obtained have been reported in the table below. All cases fall within the range of unproblematic values, and we concluded that DFT is reliable for the description of this reaction mechanism.

| **Table S2** B_1_ and A_λ_ diagnostics on relevant transition structures and steps of the migration and cyclization pathways for MNI-Ac. The structure numbers refer to **Figure S5** above. | |
| --- | --- |
| **Structure^a^** | **A_λ_ diagnostics** |
| Migration TS (**S15**) | 0.118 |
| Cyclization TS (**S16)** | 0.115 |
| Ring opening TS (**S18**) | 0.115 |
| Leaving group departure TS (**S20**) | 0.121 |
| **Structure^a^** | **B_1_ diagnostics^b^** |
| Formation of **S19** from **S14** through **S15** | 8.36 |
| Formation of **S17** (R-Chair) from **S14** | 3.02 |
| Formation of **S19** from **S17** | 5.34 |
| Formation of **S21** from **S19** | 8.85 |
| ^a^ Refer to **Figure S5** to identify the structures from their numbers. ^b^ units are kcal/mol | |

**Section S9: Reaction Mechanisms of MNI-Ac Involving Cyclization.**

**The Cyclization Pathway–D (CP–D).**

According to the CP–D mechanism (**Supplementary Figure S6**, which we will compare to **Supplementary Figure S3** for MDNI-Ac) once **S17** is formed it can undergo hydrogen atom abstraction through **S24**, which requires 25.1 kcal/mol. This step has the highest energy requirement for MNI-Ac, and therefore it is the less likely to happen. The following cyclic intermediate **S25** opens up through breakage of the N­–O bond (**S26**), followed by the departure of the leaving group (**S27**). Dis-excitation to the singlet surface (**S22**) and tautomerization (**S23**) are the same as in the mechanism discussed previously. The highest activation energy for this pathway is slightly higher in energy than the corresponding step for MDNI-Ac by ~3.0 kcal/mol. Otherwise, as we saw for all the other pathways, the differences between MDNI-Ac and MNI-Ac are minimal.

| 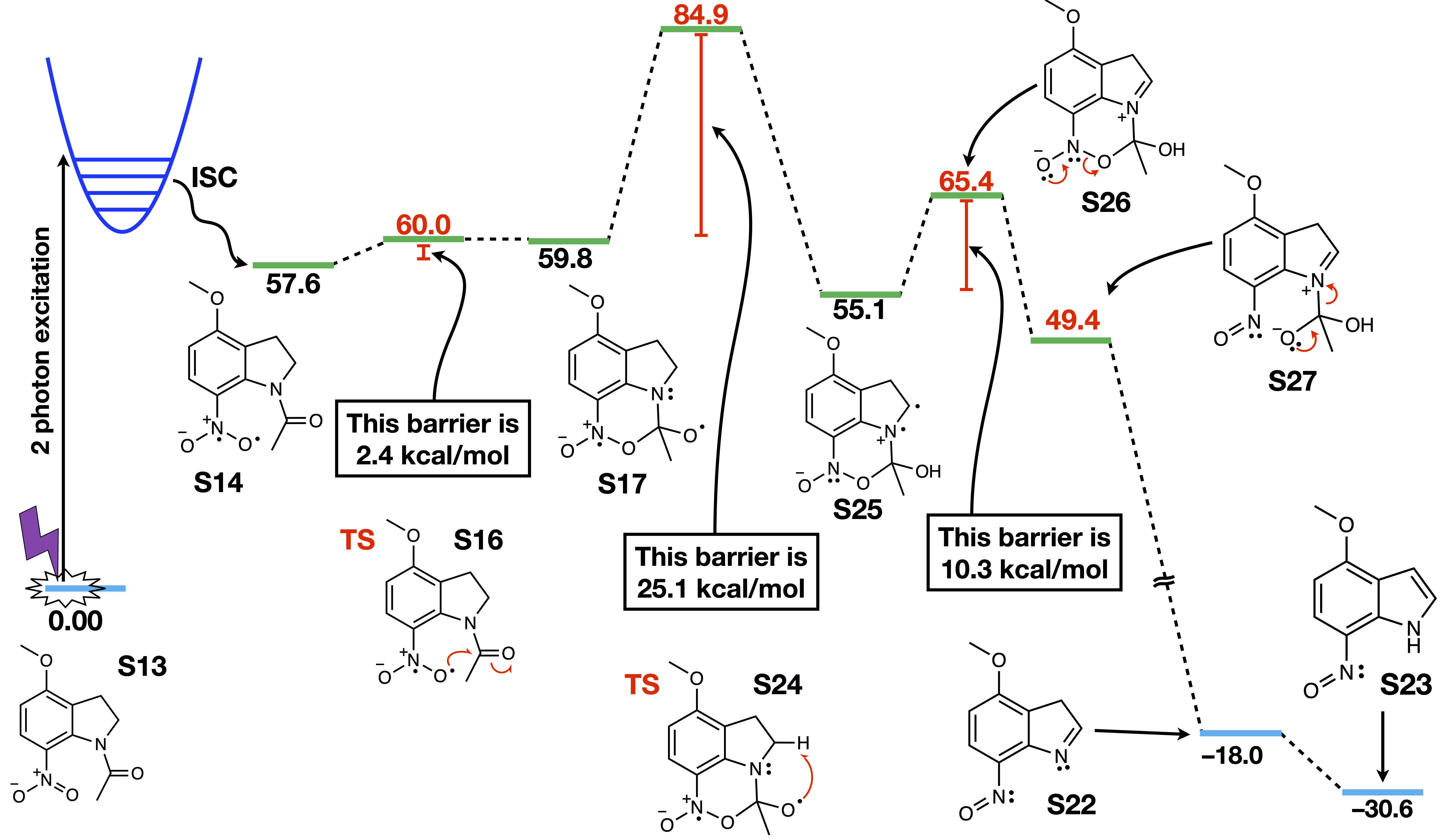 |
| --- |
| **Supplementary Figure S6** The CP–D reaction mechanism for MNI-Ac. The triplet surface is reported in green, while the singlet surface is in blue. The reported numerical values are Gibbs free energies calculated with respect to MNI-Ac in the singlet state (structure **S17**) and they have been obtained at the ωB97X-D/def2-TZVP level of theory in water (C-PCM). All values are in kcal/mol. |

**Section S10: Reaction Mechanisms of MNI-Ac Involving Cyclization. Pathway CP–B and Singlet Reactivity.**

As last pathway (CP–B), we analyze the outcome of breaking the N–O bond of the cyclic intermediate **S17**. The cyclic intermediate **S17** is formed in the same way as for the previous pathways (**Supplementary Figures S5 and S6**). In this case, the intermediate opens up after overcoming an activation barrier of 22.6 kcal/mol (structure **S28**, **Supplementary Figure S7**). This value is almost identical to the 22.2 kcal/mol reported for MDNI-Ac, confirming one more time that the substitution pattern on the cage does not influence the reactivity of the molecule on the triplet surface. After locating the transition structure **S28**, we could not locate the corresponding intermediate. As for MDNI-Ac, the structure would revert to the cyclic intermediate **S17** during geometry optimization. In addition, this high-energy pathway does not lead to an experimentally observed intermediate, so we did not explore its reactivity any further.

The hydrolysis side-reaction has been taken into account as well. This process requires 50.3 kcal/mol (structure **S29**), compared to the 43.7 kcal/mol required by MDNI-Ac (**S12**). This process is the least favored, and it does not represent a problem for MNI-Ac.

Last, we located the cyclization transition structure on the singlet surface for MNI-Ac as well. The cyclization process for MNI-Ac requires 39.0 kcal/mol, which is 3.9 kcal/mol higher than MDNI-Ac.

| 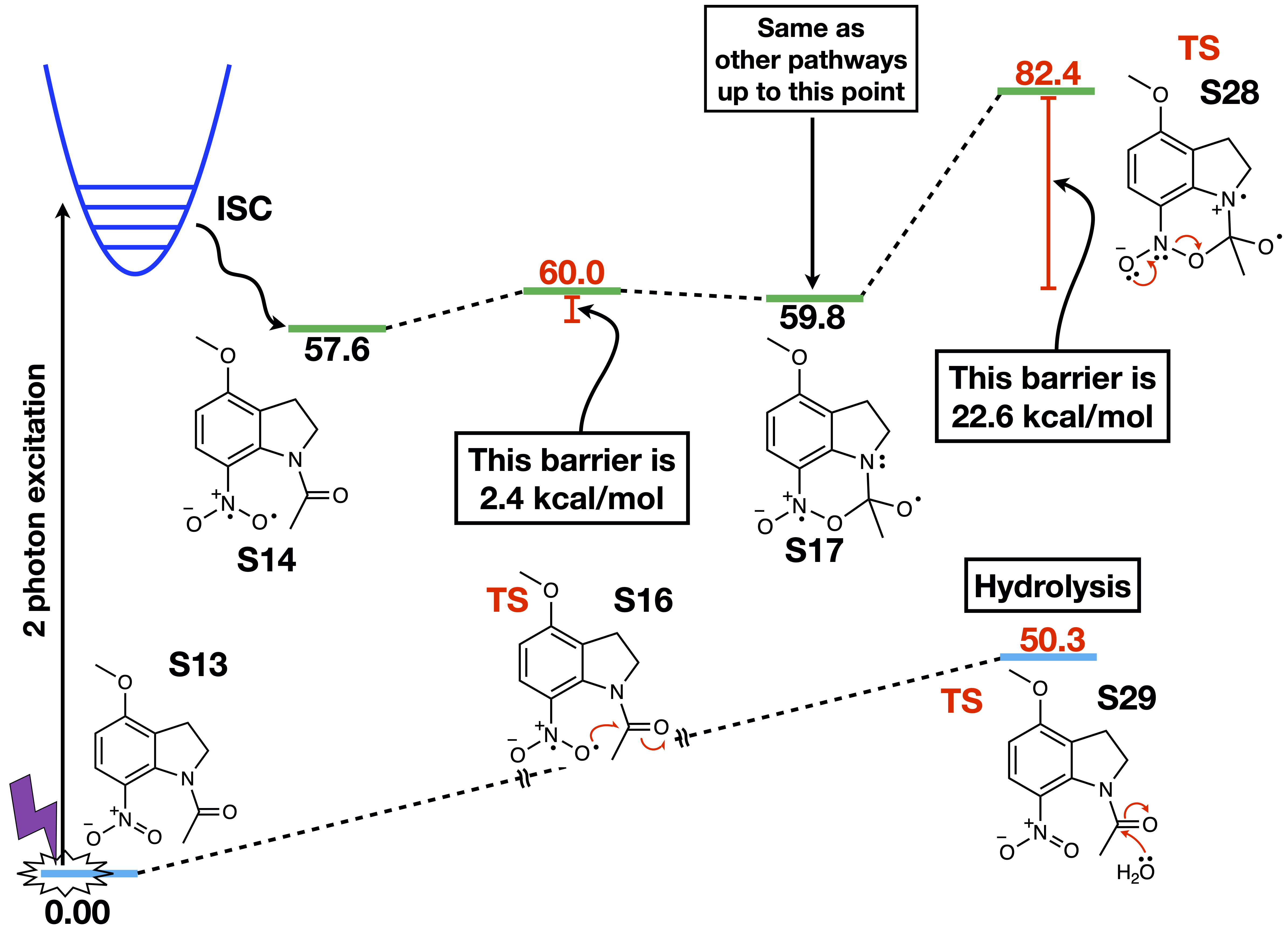 |
| --- |
| **Supplementary Figure S7** The CP–B mechanism (upper section) and the hydrolysis mechanism (lower section) for MNI-Ac. The triplet surface is reported in green, while the singlet surface is in blue. The reported numerical values are Gibbs free energies calculated with respect to MNI-Ac in the singlet state (structure **S13**) and they have been obtained at the ωB97X-D/def2-TZVP level of theory in water (C-PCM). All values are in kcal/mol. |

**Section S11: Compounds containing glutamate.**

As validation of our findings, we investigated the first step of the CP–C and MP mechanisms for the uncaging of glutamate in addition to the already reported uncaging of MDNI-Ac and MNI-Ac. Specifically, we investigated the MDNI-Glu, MNI-Glu, 𝛼-MDNI-Glu, and 𝛼-MNI-Glu structures. As explained in the main text, the only difference among these structures lies in the way the glutamate is bound to the cage. In the case of MDNI-Glu and MNI-Glu, the caging agent is bound to the terminal carbonyl group of the glutamate side chain, while in the case of 𝛼-MDNI-Glu and 𝛼-MNI-Glu, the cage is bound to the carbonyl group linked to the 𝛼-carbon of the amino acid. We report the relative Gibbs free energies calculated with the ωB97X-D functional and the def2-TZVP basis set using the compounds in the singlet state as references in **Table S3**. We refer to the Excel file “results_Glu.xlsx” for an overview of the results obtained with the other functionals. For these molecules, we could not obtain results with the double-hybrid DSD-PBEP86-D3(BJ) functional because it is unfortunately too expensive for our computational capabilities.

Our results show that there is competition between the cyclization and migration pathways for the compounds containing glutamate as well, but the differences in the two mechanisms are negligible. These results confirm our findings for the acetate case that we presented in the main text and above. Overall, the energy barriers are very close to each other, with the minimum being 1.1 kcal/mol for 𝛼-MDNI-Glu and the maximum being 4.3 kcal/mol for 𝛼-MNI-Glu. The other two compounds are instead identical, with a difference in reactivity of 2.3 (MDNI-Glu) and 2.6 (MNI-Glu) kcal/mol respectively. Our findings confirm that the difference in reactivity does not justify the quantum yield, in contrast to what has been previously reported.^20^ Instead, the quantum yields for these compounds can be justified by looking at the responsiveness of these molecules towards light, as reported in **Table 2** of the main text.

| **Table S3** Gibbs free energies relative to the different compounds in the singlet state obtained with the ωB97X-D/def2-TZVP functional and basis set. The number in parenthesis represent the activation energies for the relevant transition structures calculated with respect to the compounds in the triplet state (column 2). All values are in kcal/mol. | | | | |
| --- | --- | --- | --- | --- |
| **Compound** | **Relative energy, Triplet^a,b^** | **Relative energy,**  **Cyclization TS^a,b^** | **Relative energy,**  **Migration TS^a,b^** | **Migration/Cyclization**  **difference^b^** |
| MDNI-Glu | 54.1 | 58.3 (4.2)^c^ | 60.6 (6.5)^c^ | 2.3 |
| MNI-Glu | 53.2 | 61.4 (8.2)^c^ | 64.0 (10.8)^c^ | 2.6 |
| 𝛼-MDNI-Glu | 52.5 | 57.8 (5.3)^c^ | 58.9 (6.4)^c^ | 1.1 |
| 𝛼-MNI-Glu | 51.9 | 57.6 (5.7)^c^ | 61.9 (10.0)^c^ | 4.3 |
| ^a^The relative energies have been calculated with respect to the compound in the singlet surface  ^b^Values in kcal/mol.  ^c^ The values in parenthesis represent activation energies calculated with respect to the compounds in the triplet state (column 2) | | | | |

**Section S12: More details on the TD-DFT calculations.**

We calculated the excitation energies of the first ten excited singlet states and the first ten excited triplet states with time-dependent density functional theory (TD-DFT, refer to **Sections S13** and **S14** below for further details on the calculations, and for a Q-Chem sample input file). We found three Singlet → Singlet transitions in the experimentally relevant region between 250 and 500 nm, as shown in the spectra of **Figure 7** in the main text. The orbital character analysis of each transition reveals that they involve either the LUMO, or the LUMO+1 orbital. These transitions for (α-)MNI-Glu have a ^1^(π → π^*^) character for S_0_ → S_1_, a ^1^(σ → π^*^) character for S_0_ → S_2_, and a^1^(σ → π^*^) character for S_0_ → S_3_. For (α-)MDNI-Glu, the transitions have a ^1^(π → π^*^) character for S_0_ → S_1_, a ^1^(σ → π^*^) character for S_0_ → S_2_, and a ^1^(π → π^*^) character for S_0_ → S_3_. We repeated the same analysis for all the triplet states lying below the third excited singlet states for each molecule. As **Table 2** of the main text and **Table S4** show, (α-)MDNI-Glu has access to seven triplet states, while (α-)MNI-Glu has access to six. The numerical values for the singlet and triplet excited states energies are reported in **Tables 2** of the main text for MDNI-Glu and MNI-Glu, and **Table S5** below for α-MDNI-Glu and α-MNI-Glu. The same data can also be found in the Excel file “results_Glu.xlsx” in the sheet called “TD-DFT_Calculations”.

| **Table S4**: Details of the excitation types for the singlet and triplet states in the experimental range (250 – 500 nm) together with the excitation energy (in eV) for 𝛼-MNI-Glu and 𝛼-MDNI-Glu. The relevant data for MDNI-Glu and MNI-Glu are reported in the main text (**Table 2**). | | | | | | | | |
| --- | --- | --- | --- | --- | --- | --- | --- | --- |
| **Molecule** | **State** | **Energy (eV)** | | **Orbital Character** | **Molecule** | **State** | **Energy (eV)** | **Transition Character** |
| 𝛼-MNI-Glu | S_1_ | 3.61 | | ^1^(π → π^*^) | 𝛼-MDNI-Glu | S_1_ | 3.62 | ^1^(π → π^*^) |
|  | S_2_ | 3.98 | | ^1^(σ → π^*^) |  | S_2_ | 3.91 | ^1^(σ → π^*^) |
|  | S_3_ | 4.31 | | ^1^(π → π^*^) |  | S_3_ | 4.01 | ^1^(σ → π^*^) |
|  | T_1_ | 3.01 | | ^3^(π → π^*^) |  | T_1_ | 2.95 | ^3^(π → π^*^) |
|  | T_2_ | 3.16 | | ^3^(π → π^*^) |  | T_2_ | 3.28 | ^3^(π → π^*^) |
|  | T_3_ | 3.53 | | ^3^(σ → π^*^) |  | T_3_ | 3.43 | ^3^(π → π^*^) |
|  | T_4_ | 3.63 | | ^3^(σ → π^*^) |  | T_4_ | 3.47 | ^3^(σ → π^*^) |
|  | T_5_ | 4.02 | | ^3^(π → π^*^) |  | T_5_ | 3.48 | ^3^(σ → π^*^) |
|  | T_6_ | 4.20 | | ^3^(σ → π^*^) |  | T_6_ | 3.69 | ^3^(σ → π^*^) |
|  |  | | N/A | |  | T_7_ | 3.71 | ^3^(σ → π^*^) |

As reported in the main text, we found a qualitative agreement with El-Sayed’s rule for two transitions for (𝛼-)MDNI-Glu and one transition for (𝛼-)MNI-Glu. The orbital characters for each transition are reported in **Table S5**.

| **Table S5**: Details of the excitation types for the triplet states coupled with the first singlet state (S_1_). They have been obtained by combining the transition characters from **Table S4**. | | |
| --- | --- | --- |
| **Molecule** | **State** | **Excitation type** |
| MNI-Glu | S_1_,T_1_ | ^1^(π → π^*^) ⇝ ^3^(π → π^*^) |
|  | S_1_,T_2_ | ^1^(π → π^*^) ⇝ ^3^(π → π^*^) |
|  | S_1_,T_3_ | ^1^(π → π^*^) ⇝ ^3^(σ → π^*^) |
| MDNI-Glu | S_1_,T_1_ | ^1^(π → π^*^) ⇝ ^3^(π → π^*^) |
|  | S_1_,T_2_ | ^1^(π → π^*^) ⇝ ^3^(π → π^*^) |
|  | S_1_,T_3_ | ^1^(π → π^*^) ⇝ ^3^(π → π^*^) |
|  | S_1_,T_4_ | ^1^(π → π^*^) ⇝ ^3^(σ → π^*^) |
|  | S_1_,T_5_ | ^1^(π → π^*^) ⇝ ^3^(σ → π^*^) |
| 𝛼-MNI-Glu | S_1_,T_1_ | ^1^(π → π^*^) ⇝ ^3^(π → π^*^) |
|  | S_1_,T_2_ | ^1^(π → π^*^) ⇝ ^3^(π → π^*^) |
|  | S_1_,T_3_ | ^1^(π → π^*^) ⇝ ^3^(σ → π^*^) |
| 𝛼-MDNI-Glu | S_1_,T_1_ | ^1^(π → π^*^) ⇝ ^3^(π → π^*^) |
|  | S_1_,T_2_ | ^1^(π → π^*^) ⇝ ^3^(π → π^*^) |
|  | S_1_,T_3_ | ^1^(π → π^*^) ⇝ ^3^(π → π^*^) |
|  | S_1_,T_4_ | ^1^(π → π^*^) ⇝ ^3^(σ → π^*^) |
|  | S_1_,T_5_ | ^1^(π → π^*^) ⇝ ^3^(σ → π^*^) |

**Section S13: Computational Details.**

All calculations on the singlet and triplet surfaces have been performed with the Gaussian16 program.^22^ We used the B3LYP-D3(BJ) functional^23–26^ with the def2-SVPD basis set^27–30^ for all geometry optimization calculations. Instead, we used the M11,^4^ MN15,^6^ ωB97X-D,^3^ LC-ωHPBE,^1^ and CAM-B3LYP^2^ functionals with the def2-TZVP basis set^27^ for frequency calculations. In addition, we employed the def2-TZVP basis set for single-point energy calculations with the DSD-PBEP86-D3(BJ)^7–9^ double-hybrid functional with Gaussian and the ωB97M-V functional as implemented in the Q-Chem program.^31^ We did not perform frequency calculations with this two functionals because they are computationally too expensive. We corrected the SCF energies with the average Gibbs free energy corrections coming from all the other functionals to remove any bias, as detailed below. In all cases, a Lebedev integration grid of 99 radial and 590 angular points —abbreviated as (99,590)— has been used. All structures have been characterized either as minima (the eigenvalues of the Hessian matrix are all positive) or transition structures (there is one negative eigenvalue in the Hessian matrix). Unfortunately, the hydrolysis transition structure for MDNI-Ac and MNI-Ac, and the cyclization structure for 𝛼-MDNI-Glu have two negative eigenvalues, and we had to correct the resulting Gibbs free energy values by excluding the entropy contribution due to the additional negative eigenvalue(s). For the two hydrolysis transition states, we attribute the difficulty in converging the calculations to the flatness of the potential energy surface. The 𝛼-MDNI-Glu structure is instead a transition state at the B3LYP-D3(BJ)/def2-SVPD level of theory, and the second negative eigenvalue arises because we are using a different functional and basis set for thermochemistry calculations. In all cases, we corrected the resulting Gibbs free energy values, but we report the corrected and uncorrected values, as well as a detail of the negative eigenvalues, in the Excel files “results_MDNI.xlsx”, “results_MNI.xlsx”, and “results_Glu.xlsx”. In all cases, the nature of these structures does not affect our conclusions.

Time-Dependent Density Functional Theory calculations have been performed on the geometries optimized on the singlet surface. We used the ωB97X-D functional for its good performance on excited states^12–15^ and because it is the best in reproducing the experimental UV-VIS spectrum for both MDNI and MNI (see **Figure 7** in the main text). The def2-TZVP basis set^27^ was used for these calculations as well, as shown in the sample input file. We also employed the Tamm-Dancoff approximation to speed up these calculations without sacrificing the accuracy of the results.^32^ We accounted for ten excited singlet states and ten excited triplet states. All calculations (geometry optimizations, frequency calculations and TD-DFT) have been performed in water using the Conductor-like Polarizable Continuum Model (C-PCM) framework.^33,34^

The analysis of the orbital character of the excitations has been performed with the IQ-mol program,^35^ by reading the formatted check-point files (.fchk) obtained with the Q-Chem program. The .fchk files for all the molecules considered are available in our group’s GitHub page (https://github.com/peverati/MDNI-MNI_Uncaging_Mechanism).

Multi-Reference Diagnostics.

The B_1_ diagnostic^18^ of Schultz, Zhao and Truhlar takes into account the bond dissociation energies and the number of bonds broken in a reaction step. We took all the relevant steps of the two main pathways into account, as detailed in **Sections** **S4** and **S8**. Here, we use the migration step in the MDNI-Ac reaction as an example. In order to calculate the B_1_ value for this step, we need to calculate the bond dissociation energy (BDE) with BLYP^23,24^ and B1LYP^23,24,36,37^ and then we need to divide it by the number of bonds broken *n*.

B_1_ = [BDE(BLYP) – BDE(B1LYP)]/*n*

In both steps considered, we only break one bond (*n*=1), so the B_1_ value is simply equal to the difference between the BDEs calculated with the two functionals in terms of electronic energies (EE):

BDE[B(1)LYP] = (EE of the nitronic anhydride – EE of MDNI in the triplet)

With the A_λ_ diagnostic^19^ we took into account the transition states geometries. The A_λ_ diagnostic is defined as

$$A_{\lambda}=\frac{1 - \frac{TAE(\lambda)}{TAE(0)}}{\lambda}$$

where TAE stands for total atomization energy and λ represents the fraction of Hartree-Fock (HF) exchange included in the definition of a functional. We used the functionals BLYP and B1LYP because they correlate best with wave-function-theory-based diagnostics^19^ and for consistency with the B_1_ case. Since B1LYP includes 25% of HF exchange, which corresponds to λ = 0.25, the equation above reduces to:

$$A_{\lambda}=4*(1-\frac{\mathrm{TAE}\left( B1LYP \right)}{\mathrm{TAE}\left( \mathrm{BLYP} \right)})$$

For all these calculations, we used the Gaussian program and the BLYP and B1LYP functionals. For the B_1_ diagnostic, we used the MG3^38–43^ basis set as reported in the original publication,^18^ while for the A_λ_ diagnostic we used the cc-pVTZ basis set,^44^ as recommended by Martin and coworkers.^19^ Numerical results are reported in the Excel files.

**Section S14: Detailed Description of the Procedure Used to Correct the Structures with More than One Negative Eigenvalue.**

As stated previously, we did not perform frequency calculations with the DSD-PBEP86-D3(BJ) functional due to the prohibitive cost of this kind of calculation. We did not perform frequency calculations with the ωB97M-V functional because it is not (yet) possible to perform this type of calculations analytically with the Q-Chem code. For this two functionals, we corrected the electronic energies by using the average of the Gibbs free energy contributions calculated with the other functionals. All the numerical results are presented in detail in the three Excel files, but we will use the MDNI-Ac molecule in the singlet state (structure **16** in **Figure 6** of the main text) as an example. The Gibbs free energy contribution of each functional is reported in **Table S6**.

| **Table S6**: Gibbs free energy contributions (in Hartrees) as calculated with different functionals and the def2-TZVP basis set. The average column shows the contribution added to the electronic energies calculated with the DSD-PBEP86-D3(BJ) and the ωB97M-V functionals using the same basis set. | | | | | |
| --- | --- | --- | --- | --- | --- |
| **ωB97X-D** | **M11** | **MN15** | **LC-ωHPBE** | **CAM-B3LYP** | **Average** |
| 0.18526 | 0.18371 | 0.18391 | 0.18540 | 0.18767 | 0.18512 |

For the ωB97M-V functional, the (absolute) electronic energy of structure 16 is –1041.30920 Hartrees, and the corresponding absolute value of Gibbs free energy is (–1041.30920 + 0.18512) = –1041.12463 Hartrees. The other values have been obtained in the same way.

For the hydrolysis transition structures of MNI-Ac and MDNI-Ac, we had to perform constrained geometry optimizations, with different algorithms (with both the Gaussian and Q-Chem code) to locate the corresponding geometries. Unfortunately, it has not always been possible to locate transition structures with only one negative eigenvalue due to the flatness of the potential energy surface in certain regions. In order to account for a correct value in the Gibbs free energy corrections, we removed the entropic contribution associated with the unwanted negative eigenvalues and we used the corrected value to calculate the energy differences and their average values. We use the hydrolysis structure for MDNI-Ac (**S12**) and the ωB97X-D functional as an example. The values reported in **Table S7** can also be found in the Excel file called “results_MDNI.xlsx”.

| **Table S7**: Entropic contributions and corrections to the reported Gibbs Free energy value for Structure **S12** as calculated at the ωB97X-D/def2-TZVP level of theory. | | | | | | |
| --- | --- | --- | --- | --- | --- | --- |
| **Total Entropy^a^** | **Vibration 1 Entropy^a^** | **Vibration 2 Entropy^a^** | **Electronic Energy^b^** | **Uncorrected contribution^b^** | **Corrected contribution^b^** | **Reported Free Energy^b^** |
| 130.984 | 4.262 | 3.489 | –1117.673193 | 0.209969 | 0.211627 | –1117.461566 |
| ^a^ Units are cal mol^–1^ K^–1^; ^b^units are Hartrees | | | | | | |

A detail of all the negative eigenvalues found, and their respective corrections is reported in the Excel files. The structures involved belong to the secondary mechanisms, specifically we refer to the hydrolysis structures for both MDNI-Ac and MNI-Ac (Structures **S12** and **S29**), and the cyclization structure for 𝛼-MDNI-Glu. In all cases, the unwanted eigenvalues do not affect the eigenvalue of interest, as the respective changes are minimal.

**Section S15: Sample Input Files.**

Gaussian16.

The following route has been used for all geometry optimizations. The def2-SVPD basis set is non-standard, so it has to be read using the Gen keyword in Gaussian 16.

#P Opt B3LYP/Gen Integral=UltraFineGrid SCF=(NoSymm,MaxCyc=200,Tight) IOp(1/33=1) SCRF(CPCM,Solvent=Water) EmpiricalDispersion=GD3BJ

The following route has been used for all frequency calculations using the geometries optimized at the B3LYP-D3(BJ)/def-SVPD level of theory. ωB97X-D is used as an example.

#P Freq GFInput wB97XD/def2TZVP SCF=(MaxCyc=250,NoSymm,Tight) Integral(Grid=UltraFine) IOp(1/33=1,6/7=3) SCRF(CPCM,Solvent=Water)

Q-Chem.

For the TD-DFT calculations, we used this input file.

$Rem

SYMMETRY False

SYM_IGNORE True

BASIS def2-TZVP

METHOD wB97X-D

MAX_SCF_CYCLES 75

SCF_ALGORITHM GDM

SCF_CONVERGENCE 8

XC_GRID 000099000590

SOLVENT_METHOD PCM

MEM_TOTAL 20000

CIS_N_ROOTS 10

CIS_CONVERGENCE 8

CIS_SINGLETS True

CIS_TRIPLETS True

GUI 2

$End

$PCM

Theory CPCM

Method SWIG

Solver Inversion

Radii Bondi

$End

$Solvent

Dielectric 78.39

$End

**Section S16: References.**

(1) Henderson, T. M.; Izmaylov, A. F.; Scalmani, G.; Scuseria, G. E. Can Short-Range Hybrids Describe Long-Range-Dependent Properties? *J. Chem. Phys.* **2009**, *131* (4), 044108.

(2) Yanai, T.; Tew, D. P.; Handy, N. C. A New Hybrid Exchange–Correlation Functional Using the Coulomb-Attenuating Method (CAM-B3LYP). *Chem. Phys. Lett.* **2004**, *393*, 51–57.

(3) Chai, J.-D.; Head-Gordon, M. Long-Range Corrected Hybrid Density Functionals with Damped Atom-Atom Dispersion Corrections. *Phys. Chem. Chem. Phys.* **2008**, *10* (44), 6615–6620.

(4) Peverati, R.; Truhlar, D. G. Improving the Accuracy of Hybrid Meta-GGA Density Functionals by Range Separation. *J. Phys. Chem. Lett.* **2011**, *2*, 2810–2817.

(5) Mardirossian, N.; Head-Gordon, M. ωB97M-V: A Combinatorially Optimized, Range-Separated Hybrid, Meta-GGA Density Functional with VV10 Nonlocal Correlation. *J. Chem. Phys.* **2016**, *144* (21), 214110.

(6) Yu, H. S.; He, X.; Li, S. L.; Truhlar, D. G. MN15: A Kohn–Sham Global-Hybrid Exchange–Correlation Density Functional with Broad Accuracy for Multi-Reference and Single-Reference Systems and Noncovalent Interactions. *Chem. Sci.* **2016**, *7* (8), 5032–5051.

(7) Grimme, S.; Ehrlich, S.; Goerigk, L. Effect of the Damping Function in Dispersion Corrected Density Functional Theory. *J. Comput. Chem.* **2011**, *32* (7), 1456–1465.

(8) Kozuch, S.; Martin, J. M. L. DSD-PBEP86: In Search of the Best Double-Hybrid DFT with Spin-Component Scaled MP2 and Dispersion Corrections. *Phys. Chem. Chem. Phys.* **2011**, *13* (45), 20104.

(9) Kozuch, S.; Martin, J. M. L. Spin-Component-Scaled Double Hybrids: An Extensive Search for the Best Fifth-Rung Functionals Blending DFT and Perturbation Theory. *J. Comput. Chem.* **2013**, *34* (27), 2327–2344.

(10) Goerigk, L.; Hansen, A.; Bauer, C.; Ehrlich, S.; Najibi, A.; Grimme, S. A Look at the Density Functional Theory Zoo with the Advanced GMTKN55 Database for General Main Group Thermochemistry, Kinetics and Noncovalent Interactions. *Phys. Chem. Chem. Phys.* **2017**, *19* (48), 32184–32215.

(11) Mardirossian, N.; Head-Gordon, M. Thirty Years of Density Functional Theory in Computational Chemistry: An Overview and Extensive Assessment of 200 Density Functionals. *Mol. Phys.* **2017**, *115* (19), 2315–2372.

(12) Caricato, M.; Trucks, G. W.; Frisch, M. J.; Wiberg, K. B. Electronic Transition Energies: A Study of the Performance of a Large Range of Single Reference Density Functional and Wave Function Methods on Valence and Rydberg States Compared to Experiment. *J. Chem. Theory Comput.* **2010**, *6* (2), 370–383.

(13) Isegawa, M.; Peverati, R.; Truhlar, D. G. Performance of Recent and High-Performance Approximate Density Functionals for Time-Dependent Density Functional Theory Calculations of Valence and Rydberg Electronic Transition Energies. *J. Chem. Phys.* **2012**, *137*, 244104.

(14) Laurent, A. D.; Jacquemin, D. TD-DFT Benchmarks: A Review. *Int. J. Quantum Chem.* **2013**, *113* (17), 2019–2039.

(15) Migliore, A. How To Extract Quantitative Information on Electronic Transitions from the Density Functional Theory “Black Box.” *J. Chem. Theory Comput.* **2019**, *15* (9), 4915–4923.

(16) Fedoryak, O. D.; Sul, J.-Y.; Haydon, P. G.; Ellis-Davies, G. C. R. Synthesis of a Caged Glutamate for Efficient One- and Two-Photon Photorelease on Living Cells. *Chem. Commun.* **2005**, No. 29, 3664.

(17) Morrison, J.; Wan, P.; Corrie, J. E. T.; Papageorgiou, G. Mechanisms of Photorelease of Carboxylic Acids from 1-Acyl-7-Nitroindolines in Solutions of Varying Water Content. *Photochem. Photobiol. Sci.* **2002**, *1* (12), 960.

(18) Schultz, N. E.; Zhao, Y.; Truhlar, D. G. Density Functionals for Inorganometallic and Organometallic Chemistry. *J. Phys. Chem. A* **2005**, *109* (49), 11127–11143.

(19) Fogueri, U. R.; Kozuch, S.; Karton, A.; Martin, J. M. L. A Simple DFT-Based Diagnostic for Nondynamical Correlation. *Theor. Chem. Acc.* **2013**, *132* (1), 1291.

(20) Pálfi, D.; Chiovini, B.; Szalay, G.; Kaszás, A.; Turi, G. F.; Katona, G.; Ábrányi-Balogh, P.; Szőri, M.; Potor, A.; Frigyesi, O.; Lukácsné Haveland, C.; Szadai, Z.; Madarász, M.; Vasanits-Zsigrai, A.; Molnár-Perl, I.; Viskolcz, B.; Csizmadia, I. G.; Mucsi, Z.; Rózsa, B. High Efficiency Two-Photon Uncaging Coupled by the Correction of Spontaneous Hydrolysis. *Org. Biomol. Chem.* **2018**, *16* (11), 1958–1970.

(21) Cohen, A. D.; Helgen, C.; Bochet, C. G.; Toscano, J. P. The Mechanism of Photoinduced Acylation of Amines by *N* -Acyl-5,7-Dinitroindoline as Determined by Time-Resolved Infrared Spectroscopy. *Org. Lett.* **2005**, *7* (14), 2845–2848.

(22) Frisch, M. J.; Trucks, G. W.; Schlegel, H. B.; Scuseria, G. E.; Robb, M. A.; Cheeseman, J. R.; Scalmani, G.; Barone, V.; Petersson, G. A.; Nakatsuji, H.; Li, X.; Caricato, M.; Marenich, A. V.; Bloino, J.; Janesko, B. G.; Gomperts, R.; Mennucci, B.; Hratchian, H. P.; Ortiz, J. V.; Izmaylov, A. F.; Sonnenberg, J. L.; Williams-Young, D.; Ding, F.; Lipparini, F.; Egidi, F.; Goings, J.; Peng, B.; Petrone, A.; Henderson, T.; Ranasinghe, D.; Zakrzewski, V. G.; Gao, J.; Rega, N.; Zheng, G.; Liang, W.; Hada, M.; Ehara, M.; Toyota, K.; Fukuda, R.; Hasegawa, J.; Ishida, M.; Nakajima, T.; Honda, Y.; Kitao, O.; Nakai, H.; Vreven, T.; Throssell, K.; Montgomery, J. A., Jr.; Peralta, J. E.; Ogliaro, F.; Bearpark, M. J.; Heyd, J. J.; Brothers, E. N.; Kudin, K. N.; Staroverov, V. N.; Keith, T. A.; Kobayashi, R.; Normand, J.; Raghavachari, K.; Rendell, A. P.; Burant, J. C.; Iyengar, S. S.; Tomasi, J.; Cossi, M.; Millam, J. M.; Klene, M.; Adamo, C.; Cammi, R.; Ochterski, J. W.; Martin, R. L.; Morokuma, K.; Farkas, O.; Foresman, J. B.; Fox, D. J. **Gaussian 16**, *Revision A.03*; **2016**.

(23) Becke, A. D. Density-Functional Exchange-Energy Approximation with Correct Asymptotic-Behavior. *Phys. Rev. A* **1988**, *38* (6), 3098–3100.

(24) Lee, C.; Yang, W.; Parr, R. G. Development of the Colle-Salvetti Correlation-Energy Formula Into a Functional of the Electron-Density. *Phys. Rev. B* **1988**, *37*, 785–789.

(25) Becke, A. D. Density‐functional Thermochemistry. III. The Role of Exact Exchange. *J. Chem. Phys.* **1993**, *98* (7), 5648–5652.

(26) Stephens, P. J.; Devlin, F. J.; Chabalowski, C. F.; Frisch, M. J. Ab Initio Calculation of Vibrational Absorption and Circular Dichroism Spectra Using Density Functional Force Fields. *J. Phys. Chem.* **1994**, *98* (45), 11623–11627.

(27) Weigend, F.; Ahlrichs, R. Balanced Basis Sets of Split Valence, Triple Zeta Valence and Quadruple Zeta Valence Quality for H to Rn: Design and Assessment of Accuracy. *Phys. Chem. Chem. Phys.* **2005**, *7* (18), 3297–3305. https://doi.org/10.1039/b508541a.

(28) Feller, D. The Role of Databases in Support of Computational Chemistry Calculations. *J. Comput. Chem.* **1996**, *17* (13), 1571–1586.

(29) Schuchardt, K. L.; Didier, B. T.; Elsethagen, T.; Sun, L.; Gurumoorthi, V.; Chase, J.; Li, J.; Windus, T. L. Basis Set Exchange: A Community Database for Computational Sciences. *J. Chem. Inf. Model.* **2007**, *47* (3), 1045–1052.

(30) Pritchard, B. P.; Altarawy, D.; Didier, B.; Gibson, T. D.; Windus, T. L. New Basis Set Exchange: An Open, Up-to-Date Resource for the Molecular Sciences Community. *J. Chem. Inf. Model.* **2019**, *59* (11), 4814–4820.

(31) Shao, Y.; Gan, Z.; Epifanovsky, E.; Gilbert, A. T. B.; Wormit, M.; Kussmann, J.; Lange, A. W.; Behn, A.; Deng, J.; Feng, X.; Ghosh, D.; Goldey, M.; Horn, P. R.; Jacobson, L. D.; Kaliman, I.; Khaliullin, R. Z.; Kuś, T.; Landau, A.; Liu, J.; Proynov, E. I.; Rhee, Y. M.; Richard, R. M.; Rohrdanz, M. A.; Steele, R. P.; Sundstrom, E. J.; Woodcock, H. L.; Zimmerman, P. M.; Zuev, D.; Albrecht, B.; Alguire, E.; Austin, B.; Beran, G. J. O.; Bernard, Y. A.; Berquist, E.; Brandhorst, K.; Bravaya, K. B.; Brown, S. T.; Casanova, D.; Chang, C.-M.; Chen, Y.; Chien, S. H.; Closser, K. D.; Crittenden, D. L.; Diedenhofen, M.; DiStasio, R. A.; Do, H.; Dutoi, A. D.; Edgar, R. G.; Fatehi, S.; Fusti-Molnar, L.; Ghysels, A.; Golubeva-Zadorozhnaya, A.; Gomes, J.; Hanson-Heine, M. W. D.; Harbach, P. H. P.; Hauser, A. W.; Hohenstein, E. G.; Holden, Z. C.; Jagau, T.-C.; Ji, H.; Kaduk, B.; Khistyaev, K.; Kim, J.; Kim, J.; King, R. A.; Klunzinger, P.; Kosenkov, D.; Kowalczyk, T.; Krauter, C. M.; Lao, K. U.; Laurent, A. D.; Lawler, K. V.; Levchenko, S. V.; Lin, C. Y.; Liu, F.; Livshits, E.; Lochan, R. C.; Luenser, A.; Manohar, P.; Manzer, S. F.; Mao, S.-P.; Mardirossian, N.; Marenich, A. V.; Maurer, S. A.; Mayhall, N. J.; Neuscamman, E.; Oana, C. M.; Olivares-Amaya, R.; O’Neill, D. P.; Parkhill, J. A.; Perrine, T. M.; Peverati, R.; Prociuk, A.; Rehn, D. R.; Rosta, E.; Russ, N. J.; Sharada, S. M.; Sharma, S.; Small, D. W.; Sodt, A.; Stein, T.; Stück, D.; Su, Y.-C.; Thom, A. J. W.; Tsuchimochi, T.; Vanovschi, V.; Vogt, L.; Vydrov, O.; Wang, T.; Watson, M. A.; Wenzel, J.; White, A.; Williams, C. F.; Yang, J.; Yeganeh, S.; Yost, S. R.; You, Z.-Q.; Zhang, I. Y.; Zhang, X.; Zhao, Y.; Brooks, B. R.; Chan, G. K. L.; Chipman, D. M.; Cramer, C. J.; Goddard, W. A.; Gordon, M. S.; Hehre, W. J.; Klamt, A.; Schaefer, H. F.; Schmidt, M. W.; Sherrill, C. D.; Truhlar, D. G.; Warshel, A.; Xu, X.; Aspuru-Guzik, A.; Baer, R.; Bell, A. T.; Besley, N. A.; Chai, J.-D.; Dreuw, A.; Dunietz, B. D.; Furlani, T. R.; Gwaltney, S. R.; Hsu, C.-P.; Jung, Y.; Kong, J.; Lambrecht, D. S.; Liang, W.; Ochsenfeld, C.; Rassolov, V. A.; Slipchenko, L. V.; Subotnik, J. E.; Van Voorhis, T.; Herbert, J. M.; Krylov, A. I.; Gill, P. M. W.; Head-Gordon, M. Advances in Molecular Quantum Chemistry Contained in the Q-Chem 4 Program Package. *Mol. Phys.* **2015**, *113* (2), 184–215.

(32) Hirata, S.; Head-Gordon, M. Time-Dependent Density Functional Theory within the Tamm–Dancoff Approximation. *Chem. Phys. Lett.* **1999**, *314* (3–4), 291–299.

(33) Barone, V.; Cossi, M. Quantum Calculation of Molecular Energies and Energy Gradients in Solution by a Conductor Solvent Model. *J. Phys. Chem. A* **1998**, *102* (11), 1995–2001.

(34) Cossi, M.; Rega, N.; Scalmani, G.; Barone, V. Energies, Structures, and Electronic Properties of Molecules in Solution with the C-PCM Solvation Model. *J. Comput. Chem.* **2003**, *24* (6), 669–681.

(35) *IQmol, Version 2.13. Http://Iqmol.Org/*.

(36) Perdew, J. P.; Ernzerhof, M.; Burke, K. Rationale for Mixing Exact Exchange with Density Functional Approximations. *J. Chem. Phys.* **1996**, *105* (22), 9982–9985.

(37) Adamo, C.; Barone, V. Toward Reliable Adiabatic Connection Models Free from Adjustable Parameters. *Chem. Phys. Lett.* **1997**, *274* (1–3), 242–250.

(38) Krishnan, R.; Binkley, J. S.; Seeger, R.; Pople, J. A. Self-Consistent Molecular Orbital Methods. XX. A Basis Set for Correlated Wave Functions. *J. Chem. Phys.* **1980**, *72*, 650–654.

(39) Clark, T.; Chandrasekhar, J.; Spitznagel, G. W.; Schleyer, P. V. R. Efficient Diffuse Function-Augmented Basis Sets for Anion Calculations. III. The 3-21+G Basis Set for First-Row Elements, Li-F. *J. Comput. Chem.* **1983**, *4* (3), 294–301.

(40) Frisch, M. J.; Pople, J. A.; Binkley, J. S. Self‐consistent Molecular Orbital Methods 25. Supplementary Functions for Gaussian Basis Sets. *J. Chem. Phys.* **1984**, *80* (7), 3265–3269.

(41) Curtiss, L. A.; Raghavachari, K.; Redfern, P. C.; Rassolov, V.; Pople, J. A. Gaussian-3 (G3) Theory for Molecules Containing First and Second-Row Atoms. *J. Chem. Phys.* **1998**, *109* (18), 7764–7776.

(42) Fast, P. L.; Sánchez, M. L.; Truhlar, D. G. Multi-Coefficient Gaussian-3 Method for Calculating Potential Energy Surfaces. *Chem. Phys. Lett.* **1999**, *306* (5–6), 407–410.

(43) M. Tratz, C.; L. Fast, P.; G. Truhlar, D. Improved Coefficients for the Scaling All Correlation and Multi-Coefficient Correlation Methods. *PhysChemComm* **1999**, *2* (14), 70.

(44) Dunning, T. H. Gaussian Basis Sets for Use in Correlated Molecular Calculations. I. The Atoms Boron through Neon and Hydrogen. *J. Chem. Phys.* **1989**, *90* (2), 1007–1023.
